# Brain activity patterns in high-throughput electrophysiology screen predict both drug efficacies and side effects

**DOI:** 10.1038/s41467-017-02404-4

**Published:** 2018-01-15

**Authors:** Peter M. Eimon, Mostafa Ghannad-Rezaie, Gianluca De Rienzo, Amin Allalou, Yuelong Wu, Mu Gao, Ambrish Roy, Jeffrey Skolnick, Mehmet Fatih Yanik

**Affiliations:** 1 Massachusetts Institute of Technology, 77 Massachusetts Avenue, Cambridge, MA 02139 USA; 2 UZH/ETH Irchel Campus, Y17-L76, Winterthurerstrasse 190, 8057 Zürich, Switzerland; 3 Intellimedix, Cambridge, MA 02139 USA; 4 Georgia Institute of Technology, 950 Atlantic Drive, Room 2151, Atlanta, GA 30332 USA; 5 Present Address: Axcella Health, 840 Memorial Dr, Cambridge, MA 02139 USA

## Abstract

Neurological drugs are often associated with serious side effects, yet drug screens typically focus only on efficacy. We demonstrate a novel paradigm utilizing high-throughput in vivo electrophysiology and brain activity patterns (BAPs). A platform with high sensitivity records local field potentials (LFPs) simultaneously from many zebrafish larvae over extended periods. We show that BAPs from larvae experiencing epileptic seizures or drug-induced side effects have substantially reduced complexity (entropy), similar to reduced LFP complexity observed in Parkinson’s disease. To determine whether drugs that enhance BAP complexity produces positive outcomes, we used light pulses to trigger seizures in a model of Dravet syndrome, an intractable genetic epilepsy. The highest-ranked compounds identified by BAP analysis exhibit far greater anti-seizure efficacy and fewer side effects during subsequent in-depth behavioral assessment. This high correlation with behavioral outcomes illustrates the power of brain activity pattern-based screens and identifies novel therapeutic candidates with minimal side effects.

## Abstract (web summary)

One challenge in drug screening for neurological disorders is how to accurately capture disease pathology and side effects. Here, the authors developed a multi-channel recording platform based on a zebrafish genetic model of epilepsy to screen for antiepileptic drugs.

## Introduction

Drugs used to treat brain disorders have typically been discovered empirically, often using animal behavioral models. This is due in large part to the complex and highly interconnected nature of the brain, which presents substantial challenges for reductive cell culture assays and molecular target-based screens. Indeed, many widely used neurotherapeutics owe their efficacy to action at multiple molecular targets, while numerous promising compounds with potent activity in cell- or target-based assays have limited clinical utility due to off-target side effects^1, 2^. Thus, in vivo assays within the context of the intact brain are essential for neuroactive drug screening.

However, similar to in vitro assays, behavioral readouts in animal models often reduce complex neurological disorders to simple metrics that do not completely reflect underlying deficits and off-target effects. Direct high-content readouts of neural activity and brain activity patterns (BAPs) represent an attractive alternative to behavior-based screens as they may more accurately capture disease pathology, drug activity, and side effects. The technical challenges of directly monitoring in vivo brain activity in large-scale screens—combined with the high cost, low throughput, and requirement for large quantities of compounds—make the use of such advanced readouts impractical in rodent-based models. Zebrafish have recently emerged as an important new vertebrate model for CNS diseases and drug screening that may ultimately be able to meet these challenges^3–5^.

To demonstrate the power of chemical screening using brain activity pattern analysis, we developed a high-throughput local field potential (LFP) recording platform capable of monitoring brain activity simultaneously in many larvae over extended periods of time using highly sensitive intra-animal microelectrodes. LFPs reflect aggregate neural activity—including synaptic activity, action potentials, calcium spikes, and afterpotentials—within the local environment of the microelectrode, making them an attractive tool for assessing systems-level processes^6, 7^. To detect effects of drugs on brain activity patterns and brain disorders, we developed algorithms that decompose LFP signals into independent subcomponents. This allows us to monitor the in vivo consequences of neuroactive compounds on brain activity patterns in real time in order to quantify efficacy and detect potential side effects, as we confirm using an in-depth 52-metric behavioral assessment.

To validate our brain activity pattern-based approach, we conducted a screen for antiepileptic drugs (AEDs) using a clinically relevant model of epilepsy in zebrafish. Zebrafish have already shown considerable promise for studying both acute seizures and genetic epilepsies^8^. Larvae exposed to pentylenetetrazole (PTZ) and other convulsants exhibit elevated locomotor activity, seizure-like movements, and electrographic seizure activity. PTZ-induced seizures can be monitored using automated behavioral tracking systems and are suppressed by many clinically effective AEDs^9–12^. Seizure-prone lines with mutations in epilepsy-associated genes have also been characterized^13–18^. In spite of the promise of zebrafish seizure models, large-scale screens using single-metric behavioral readouts often suffer from a high false-positive rate—typically on the order of 75%—when hits are retested using electrophysiology^14, 19^.

Our screen utilized two different zebrafish lines harboring independent mutations in the sodium channel gene *SCN1A* (*scn1lab* in zebrafish). *SCN1A* encodes the pore-forming alpha subunit of the Na_V_1.1 sodium channel and is widely expressed throughout the central nervous system. *SCN1A* mutations are linked to variety of childhood epilepsies in humans^20, 21^. Dravet syndrome (DS; also known as severe myoclonic epilepsy of infancy), the most commonly reported pathology, is characterized by frequent febrile seizures that appear during the first year of life and are often refractory to treatment by standard anticonvulsants^22^. Over 1200 *SCN1A* mutations have been identified to date and the most severe clinical phenotypes correlate with complete loss-of-function mutations or point mutations in critical residues of the pore region^21, 23^. In addition to epilepsy, *SCN1A* variants have been linked to autism and rare cases of familial migraine, making it one of the most therapeutically important sodium channel genes^24, 25^. We report for the first time that *scn1lab* loss-of-function mutations in zebrafish give rise to photosensitive seizure-like activity, consistent with photosensitivity observed in many DS patients. This is the first stable genetic model of a photosensitive epilepsy that has been described in zebrafish or other common vertebrate model organisms, and therefore represents an important new tool for investigating light-triggered seizures and conducting in vivo drug screens.

Using light-triggered seizure-like locomotor activity as a simple (single-metric) behavioral readout, we screened a diverse compound collection to identify preliminary hits for in-depth characterization using our LFP platform and algorithms. In addition to spontaneous and light-triggered seizures, we observed that *scn1lab* mutants exhibit a substantial decrease in LFP pattern complexity during interictal periods. Brain activity patterns from preliminary hits were therefore assessed using a multiparametric approach: 1) seizure-like events were automatically detected using an automated seizure detection algorithm and 2) LFP pattern complexity was quantified using independent component analysis (ICA). Based on these criteria, ~20% of the hits from the preliminary simple behavioral screen proved highly effective at reducing seizure frequency and restoring LFP pattern complexity.

To verify that our top-ranked LFP candidates correlate with improved behavioral outcomes in *scn1lab* mutants, we carried out an in-depth 56-parameter behavioral assessment of all preliminary hits. Using both pathologic (ictal) and resting state (interictal) behavioral metrics, we directly compared mutant and wild-type behavioral profiles and evaluated compound effects on both seizure-driven and normal behaviors. This approach reveals a strong correlation between compounds that are effective based on brain activity patterns and those that significantly reduce seizure-associated behaviors with minimal side effects. LFP pattern analysis therefore provides a powerful tool for detecting and eliminating the many false positives produced by simple behavioral screens. Our multiparametric screening approach points toward several promising new therapeutics for DS and other epilepsies and illustrates the power of using brain activity pattern analysis for CNS drug discovery.

## Results

### A genetic model of photosensitive epilepsy

The zebrafish *scn1lab* gene (previously known as *double indemnity* or *didy*) encodes a voltage-gated sodium ion channel alpha subunit that is orthologous to the mammalian SCN alpha subfamily comprising *SCN1A*, *SCN2A*, *SCN3A*, and *SCN9A*^26, 27^. Mutations in *SCN1A—*and to a lesser extent *SCN2A*, *SCN3A*, and *SCN9A—*are associated with a variety of monogenic childhood epilepsies such as DS in humans^28–33^. A presumptive loss-of-function mutation in zebrafish *scn1lab* (*scn1lab*^*s552*^; Supplementary Fig. 1) causes spontaneous electrographic seizure-like events and elevated locomotor activity in larvae beginning at ~4 days post fertilization (dpf). Based on previous studies, exposure of *scn1lab*^*s552*^ larvae to AEDs reveals a pharmacological profile reminiscent of DS in humans^14^.

**Fig. 1:**
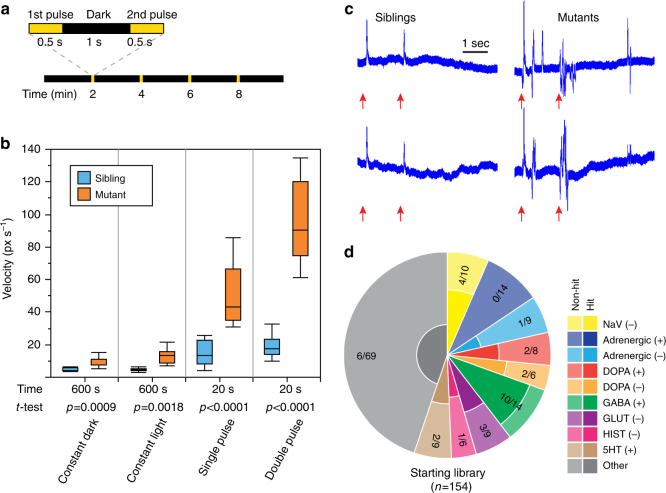
Light-induced seizures enable high-throughput screening in zebrafish larvae with *scn1lab* mutations. **a** Schematic representation of light stimulus parameters. Light stimuli are applied every 2 min in an otherwise dark environment. Each stimulus consists of two consecutive 500 ms light pulses separated by 1 s of dark. **b** Box-and-whisker plots showing mean swimming velocity in *scn1lab*^s552^ homozygous mutants (orange) and age-matched sibling controls (blue). 12 siblings and 12 mutants are used per condition. For constant dark and constant light conditions, mean swimming velocities are calculated over a full 10 min recording session. For light-triggered activity, velocities are calculated during 5 s intervals following the onset of each stimulus, resulting in a total assay time of 20 s. Tops and bottoms of each box represent the 1st and 3rd quartiles. Whiskers are drawn from the ends of the interquartile ranges (IQR) to the outermost data point that falls within ±1.5 times the IQR. The line in the middle of each box is the sample median. Statistical significance was determined by Welch’s *t*-test. **c** Representative local field potential (LFP) recordings from the forebrains of *scn1lab*^s552^ homozygous mutant larvae and age-matched sibling controls at 7 dpf in response to light stimuli. Red arrows indicate the onset of the two 500 ms light pulses. **d** Breakdown of drug classes represented in the starting library (154 compounds) and following the behavioral screen (31 compounds; *n* = 8+ larvae, each subjected to four independent light stimuli)

Photosensitive seizures, which can be triggered by flashing stimuli, bright light, or strong contrast between darkness and light, have been reported in 30–40% of patients with DS and are often associated with more severe outcomes^22, 34, 35^. We therefore sought to determine whether seizures can be triggered in *scn1lab* mutant larvae using simple visual stimuli. At 7 dpf, homozygous mutants and age-matched sibling controls (a mixture of wild-type and heterozygous larvae), were transferred to 96-well plates and locomotor activity was assessed during a 10 min recording session using an automated tracking platform capable of delivering a range of computer-controlled light stimuli. We observed elevated locomotor activity in mutants relative to siblings under both constant light and constant dark conditions (Supplementary Fig. 2a), consistent with previous reports^14^. We then stimulated the larvae by administering either (1) a single brief (500 ms) light pulse or (2) two light pulses separated by a 1 s interval. Light stimuli were administered every 2 min in an otherwise dark environment over the course of a 10 min recording session (Fig. 1a). Mutants consistently exhibited short rapid bursts of seizure-like locomotor activity commencing with the onset of the light stimulus and persisting for ~5 s (Supplementary Fig. 2b, Supplementary Movie 1). In contrast, wild-type siblings showed almost no perceptible increase in locomotor activity in response to light stimuli. In order to quantify light-triggered locomotion we calculated mean swimming velocity over a 5 s interval beginning with the onset of each light stimulus. Light-triggered locomotor activity was significantly higher in mutants than in siblings and was markedly exacerbated by the dual pulse protocol (Fig. 1b). The overall difference between mutants and sibling controls was far more pronounced in response to light stimuli than under either constant light or constant dark conditions, in spite of the fact that the total analysis interval was reduced from 10 min to only 20 s (i.e., four separate 5 s post-pulse intervals).

To verify that photosensitivity is a general feature of *scn1lab* loss-of-function rather than a unique phenotype associated with the *scn1lab*^*s552*^ missense mutation, we tested a second previously uncharacterized *scn1lab* mutant generated as part of the Zebrafish Mutation Project^36^. The *scn1lab*^*sa16474*^ allele introduces a C to A mutation at position 1386 of the *scn1lab* open reading frame, resulting in a premature stop codon at position 462 (*p*.Tyr462*)(Supplementary Fig. 1). The mutation is located in the intracellular loop between domains I and II and presumably renders the ion channel nonfunctional. Homozygous mutant *scn1lab*^*sa16474*^ larvae exhibit the same morphological phenotypes seen in *scn1lab*^*s552*^ mutants^37^, including failure to inflate swim bladders and a dark appearance due to dispersed melanosomes (Supplementary Fig. 3). Mutant larvae fail to thrive and begin to die at elevated rates relative to sibling controls beginning at approximately 13 dpf (Supplementary Fig. 3). It remains unclear if this is a secondary consequence of the swim bladder defect or a more fundamental deficit. Homozygous *scn1lab*^*sa16474*^ mutants exhibit the same seizure-like behavioral phenotypes seen with the s552 allele, including elevated locomotor activity under constant light and constant dark conditions. Photosensitivity is also present, with light pulses eliciting sudden rapid bursts of seizure-like activity (Supplementary Fig. 2b, 4).

We next assessed both *scn1lab*^*s552*^ and *scn1lab*^*sa16474*^ mutants for electrophysiological hallmarks of seizures. At 7 dpf, homozygous mutant larvae and age-matched sibling controls were embedded in low melting point agarose and forebrain LFPs were recorded over a period of 4 h. As previously reported^14^, *scn1lab*^*s552*^ mutants exhibit spontaneous high-amplitude ictal spikes when recorded under constant illumination. Spikes were observed on average every 10 ± 1.5 min in mutant larvae and were never detected in sibling controls. A similar pattern of infrequent spontaneous ictal-like electrographic discharges was observed in *scn1lab*^*sa16474*^ mutants. To verify photosensitive epilepsy, LFPs were monitored over the course of 10 min in response to our light stimulus protocol. Mutant larvae exhibited a distinctive LFP pattern in response to light stimuli, characterized by multiple high-amplitude spikes commencing shortly after the onset of each stimulus (Fig. 1c). In contrast, sibling controls from both mutant lines showed a markedly different response pattern, consisting of a single lower-amplitude spike coinciding with each light pulse (Fig. 1c) and becoming progressively diminished in amplitude with each subsequent presentation of the stimulus (Supplementary Fig. 5). Taken together, these data show for the first time that light-triggered seizures are a general feature of *scn1lab* mutations in zebrafish and establish an important new vertebrate genetic model for studying photosensitive epilepsies.

### Preliminary AED screen by light-induced locomotor activity

The ability to trigger seizures on demand in *scn1lab* mutant zebrafish using light stimuli provides a powerful tool for high-throughput AED screening. To better understand the nature of light-triggered seizures in *scn1lab* mutants and to explore the range of potentially effective therapeutics, we assembled a library consisting of 154 compounds covering specific neurotransmitter pathways and drug classes (Fig. 1d, Supplementary Data 1). Compounds chosen for screening included: (1) AEDs that are commonly used to treat patients with DS, (2) compounds with reported efficacy in treating DS based on published human studies, (3) compounds with known or suspected anticonvulsant activity in other types of epilepsy, including AEDs that are specifically contraindicated for use in DS due to interactions with the SCN1A channel, (4) known neuroactive compounds with well-characterized mechanisms of action targeting a wide spectrum of neurotransmitter pathways and covering many common classes of neuroactive drugs, and (5) compounds we identified in silico as predicted binders to human SCN1A and SCN8A based on similarity (*p*-value of 3.2 × 10^−3^) of the pocket adjacent to the voltage sensing and pore domains to a mineralocorticoid receptor pocket^38, 39^. Among the compounds identified in silico were progesterone and mifepristone.

All compounds were initially assessed for locomotor impairment and toxicity at a concentration of 100 μM. Those that exhibited overt toxicity at 4-h post exposure based on reduced/absent touch-evoked escape response were retested at lower concentrations until a maximum tolerated dose was found. Compound screening was carried out in 96-well plates and all compounds were initially tested on groups of eight homozygous mutant *scn1lab*^*s552*^ larvae (1 per well). Prior to compound application, an initial video recording was performed to establish baseline locomotor activity for each test group. Light stimuli were applied utilizing the dual-pulse parameters described previously (Fig. 1a). Locomotor activity was recorded for 5 s beginning with the onset of each light stimulus and a total of four stimuli were administered over the course of 10 min. Immediately after the baseline recording, compounds were applied directly to the wells at the indicated concentration (Supplementary Data 1) and additional recordings were performed beginning at 45 min, 2 h, and 4 h post exposure. We evaluated the effect of all compounds on abnormal light-triggered locomotor activity at each time point by calculating the mean swimming velocity of each larva in response to stimuli and normalizing to the baseline. Compounds causing a statistically significant reduction in activity (*p* < 0.05) from baseline at one or more time points were verified by rescreening on a larger pool of larvae.

We included a number of drugs with established clinical effects on DS in our library to serve as positive controls and to assist in selecting an optimal hit threshold for identifying compounds to test in detailed follow-up screening. Controls included 11 drugs that are either commonly used to treat DS or have shown efficacy in human studies (designated as “effective” in Supplementary Table 1) and 6 AEDs that have been reported to worsen seizures in patients with DS (designated “contraindicated”). A majority of the clinically effective drugs reduced abnormal locomotor activity by at least 50% at one or more of the post-exposure time points (Supplementary Table 1). In contrast, all but one of the contraindicated drugs failed to meet this criterion, indicating that photosensitivity in *scn1lab* mutant zebrafish may provide a selective readout to identify compounds appropriate for treating DS. We therefore chose a 50% reduction in abnormal light-triggered locomotor activity as our assay threshold and deemed all 31 compounds that met this criterion to be preliminary hits. These compounds were verified by screening on the *scn1lab*^*sa16474*^ line, where most gave similar results (Supplementary Table 2). Our preliminary hits included compounds with a wide variety of targets and appeared to be substantially enriched for agonists and positive allosteric modulators of γ-aminobutyric acid (GABA) receptors, particularly the GABA_A_ receptor (GABA_A_R; Fig. 1d, Supplementary Fig. 6).

### Electrophysiology screen by high-throughput LFP recording

**Fig. 2:**
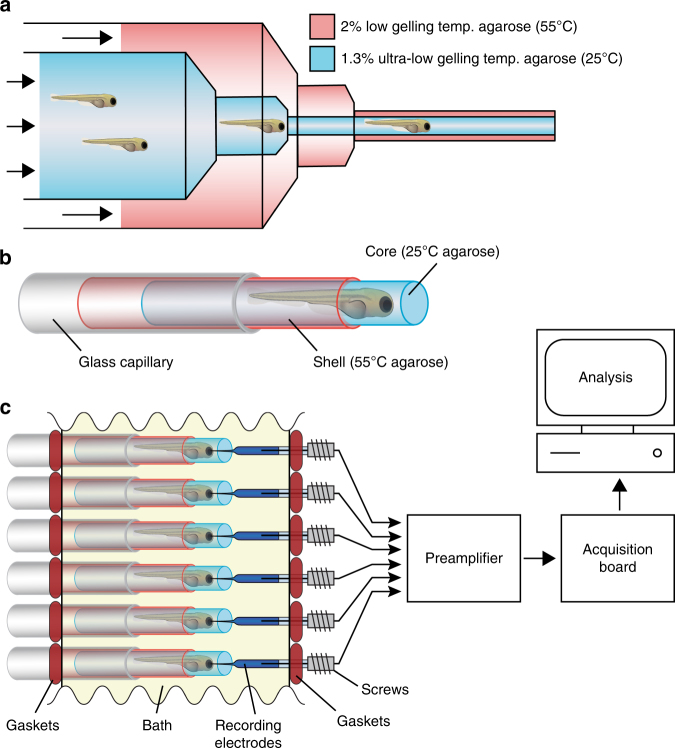
High-throughput local field potential (LFP) recording platform. **a** Zebrafish larvae are transferred to liquid 1.3% ultra-low gelling temperature agarose (25 °C) and placed inside a 20 mL syringe. The 20 mL syringe is then inserted into a 60 mL syringe filled with 2% low gelling temperature agarose (55 °C). Syringes are capped with concentric 18-gauge and 16-gauge needles, respectively, allowing both agarose solutions to be simultaneously extruded into a room temperature bath where they rapidly gel. Up to 50 larvae can be embedded in a single extrusion. **b** Diagram of a zebrafish larvae embedded in an ultra-low gelling temperature agarose core surrounded by a rigid agarose shell. Embedded larvae are loaded into glass capillaries prior to LFP recording. **c** Schematic representation of the high-throughput LFP recording platform. Embedded larvae in glass capillaries are inserted into the platform in parallel directly opposite an array of glass recording electrodes. The water-tight recording chamber bath is filled with zebrafish embryo medium and the recording electrodes are advanced into the larvae using miniaturized screws. Up to 16 larvae can be recorded simultaneously using a 16-channel preamplifier connected to a low-power digital acquisition chip

We developed an LFP recording platform capable of simultaneously monitoring many zebrafish larvae over extended periods (4+  h) to rapidly assess the in vivo effect of all 31 preliminary hits on brain activity patterns (Fig. 2). Our LFP setup consists of parallel glass capillaries that hold agar-embedded zebrafish larvae along one side of a custom-fabricated recording chamber containing the test compound of interest. On the opposite side of the recording chamber, glass recording electrodes are precisely co-centered with the agar-embedded larvae. These microelectrodes are connected to a multi-channel preamplifier, which is connected to an acquisition board. The recording electrodes are advanced forward into the forebrains of larvae using miniaturized screws. The electrical resistance and the average voltage on each electrode is monitored as it penetrates the forebrain. Advancement is halted when resistance decreases to 3 MΩ and the average noise is less than 0.2 mV RMS. In order to immobilize and precisely position non-anesthetized non-paralyzed zebrafish larvae within the glass capillaries for extended LFP recording sessions, we devised a process through which 50+ larvae can be rapidly embedded in a dual-layer agar cylinder (Fig. 2a, b; see Methods for details). When coupled to a single 16-channel preamplifier, our LFP platform allows us to obtain 4+ h recordings from up to 48 larvae in an ~12-h period. Considerably higher throughputs can be achieved simply by using a preamplifier with additional channels (e.g., 64- or 128-channels) and/or by reducing the recording time. Standard electrophysiological analysis in zebrafish typically involves relatively short recordings on the order of 10 min rather than extended 4+ h recordings. In addition, custom chips with large-scale integrated amplifiers can allow straightforward expansion of our method to industrial scale applications.

### LFP seizure score

To assess the efficacy of our preliminary hits in reducing spontaneous seizures, we developed an automated seizure detection algorithm based on methods previously used to analyze EEG signals^40^ (see Methods for details) and used it to define a seizure score. Our automated seizure detection algorithm was trained to identify seizure-like events using LFP recordings obtained from *scn1lab* mutants exposed to light stimuli as a training data set. We then used the algorithm to measure spontaneous seizure frequency in compound-treated *scn1lab* mutants. Baseline seizure frequency was first determined for each larva during a 30 min pre-exposure LFP recording. Following compound administration, spontaneous seizure frequency was measured again between 130 and 240 min post exposure (see Supplementary Table 3, seizure frequency, 240 min column). For each compound (comp), a standardized seizure score $(S_{\mathrm{comp}})$ was determined by first normalizing the post-exposure seizure frequency to the baseline frequency and then calculating $S_{\mathrm{comp}} = \left( F_{\mathrm{mut}} - F_{\mathrm{comp}} \right) / F_{\mathrm{mut}}$, where $F_{\mathrm{mut}}$ is the seizure frequency in untreated (1% DMSO) *scn1lab* mutants and $F_{\mathrm{comp}}$ is the frequency in mutants treated with the compound of interest. The seizure score therefore represents the overall improvement in seizure frequency relative to untreated mutants (i.e., untreated mutants will have a score of 0; wild-type sibling controls and compounds with 100% efficacy will have a score of 1.0; see Supplementary Table 3, “seizure score” column). In addition, at 240 min post exposure all larvae were subjected to our standard light-stimulus protocol in order to verify compound efficacy on light-triggered seizure-like activity (Supplementary Fig. 7).

### LFP complexity score

In addition to the high-amplitude spikes that characterize seizure-like events, we observed that interictal LFP activity patterns in *scn1lab* mutants appear to be considerably less complex and more stereotypic than in sibling controls. We speculated that interictal pattern structure could provide another metric to evaluate the efficacy of neuroactive compounds. In order to assess this aspect of the *scn1lab* phenotype, we utilized independent component analysis (ICA). ICA is an unsupervised analysis method for separating multivariate signals into independent subcomponents and is widely utilized for decomposition of EEG signals^41^. Typically, ICA is used to perform blind spatial filtering from multi-channel EEG recordings; however, single-channel ICA can similarly be used to perform blind temporal filtering on data from a single sensor. Single-channel ICA can accurately separate out important components from a time series provided the sources are reasonably spectrally disjoint, as has been shown to be the case for epileptic EEG data^42^. Experimental studies suggest that the spatial reach of the LFP signal is on the order of at least a few hundred micrometers^6^, a scale which encompasses a substantial portion of the larval zebrafish brain. Consequently, we assume that most of the LFP signal is the summation of transmembrane currents arising from many uncorrelated sources associated with multiple regions and neuronal subtypes. Therefore, according to the central limit theorem, the data will be approximately normally distributed. ICA exploits the fact that the rest of the superposition of independent non-Gaussian sources can be separated by optimizing the fourth moment of the input^43, 44^. To assess signal complexity, we first apply our standard 10 min light-stimulus protocol (Fig. 1a) beginning at 240 min post exposure and divide the recording into multiple 30 s intervals using a sliding time window with 80% overlap. Independent vectors are obtained as described in Methods. These vectors are then used to decompose LFP activity and calculate independent components (ICs) during a subsequent 45 min unstimulated recording session.

**Fig. 3:**
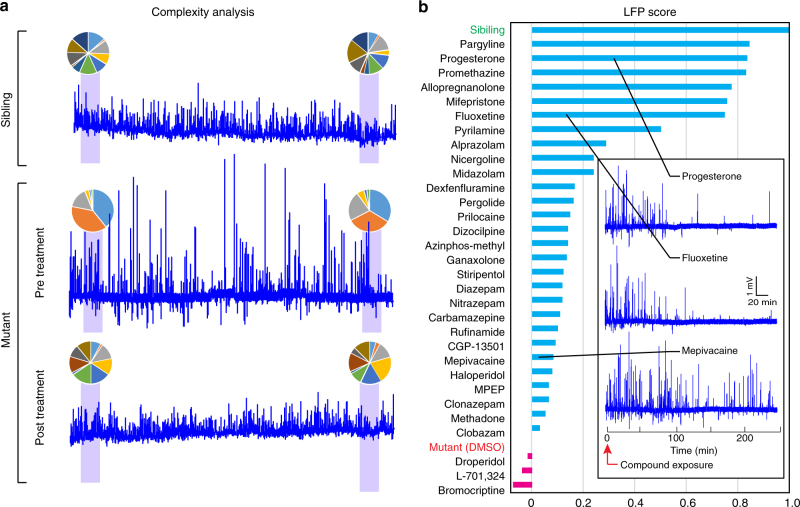
Screening neuroactive compounds using brain activity patterns. **a** LFP recordings from a representative sibling control (top), an untreated *scn1lab*^*s552*^ mutant (center) and a mutant beginning at 2 h after exposure to fluoxetine (bottom). All recordings span 4 h. Pie charts above each recording indicate the relative contribution of each independent component (IC) during the indicated 45 min interval (purple shading). Untreated mutants have low-complexity LFPs made up of only a few dominant ICs, while sibling controls and mutants treated with effective compounds have more complex LFPs that are composed of more equally dominant ICs. **b** Composite LFP scores at 4-h post treatment for all preliminary hits as well as untreated (1% DMSO) *scn1lab* mutants and age-matched sibling controls (*n* = 5–11 per compound). Inset shows representative 4 h LFP recordings from individual *scn1lab*^s552^ mutant larvae treated with the indicated compounds beginning at time = 0. The frequency of spontaneous high-amplitude seizure-like spikes diminishes in response to effective compounds (e.g., progesterone and fluoxetine)

When larvae are analyzed using our ICA approach, only a small number of ICs tend to dominate LFP traces from untreated *scn1lab* mutants (“low-complexity” LFPs), while traces from sibling controls comprise a far greater variety of ICs (“complex” LFPs; Fig. 3a). To quantify the efficacy of compounds in modulating LFP complexity, we developed an IC complexity score based on ICA at 4-h post exposure. To calculate this metric, we first rank order ICs for each compound based on intensity and then normalize all subsequent ICs to the first IC (IC_*i*_/IC_1_). Ineffective compounds and untreated mutants have low-complexity LFP signals (dominated by a few strong ICs), so normalized values drop off rapidly in the lower-ranked ICs. In contrast, complex LFPs from sibling controls and from mutants with “corrected” LFP patterns show less rapid attenuation of lower-ranked ICs (Supplementary Fig. 8a). The ability of a compound to restore LFP complexity in *scn1lab* mutants is quantified by first calculating two metrics: (1) $A_{\mathrm{mut}}$, the total area separating the IC profiles of sibling controls and untreated mutants (both in 1% DMSO; Supplementary Fig. 8b, yellow region) and (2) $A_{\mathrm{comp}}$, the total area separating the IC profiles of sibling controls and compound-treated mutants (Supplementary Table 3, total area column; specific examples shown in Supplementary Fig. 8c-h, yellow regions). For each compound, an ICA score $\left( {\mathrm{ICA}}_{\mathrm{comp}} \right)$ is then calculated as follows:

$${\mathrm{ICA}}_{\mathrm{comp}} = \left( {A}_{\mathrm{mut}} - {A}_{\mathrm{comp}} \right) / {A}_{\mathrm{mut}}$$

As with the seizure score, the ICA score reflects the overall improvement in LFP pattern complexity relative to untreated mutants (untreated mutants = 0; sibling controls=1.0; Supplementary Table 3, ICA score).

Seizure scores and ICA scores allow us to evaluate the efficacy of each compound on two scales: desirable compounds should both reduce the number of seizure-like events and restore the interictal LFP pattern to a more wild-type state. These two readouts can be combined to obtain a single composite LFP score, which provides a multiparametric indicator of overall compound efficacy. This is done by using ICA and seizure scores specify a single *XY* coordinate on a scatterplot for each compound. The composite LFP score is then calculated as follows: $\left( {\mathrm{Dist}}_{\mathrm{mut}} - {\mathrm{Dist}}_{\mathrm{comp}} \right) / {\mathrm{Dist}}_{\mathrm{mut}}$, where ${\mathrm{Dist}}_{\mathrm{mut}}$ is the Euclidean distance between sibling controls and untreated *scn1lab* mutants and ${\mathrm{Dist}}_{\mathrm{comp}}$ is the distance between sibling controls and compound-treated mutants (Supplementary Fig. 9). As with ICA and seizure scores, the closer the composite LFP score is to 1.0 the more effective the compound is at normalizing mutant brain activity patterns. Based on this score, 6 of our 31 preliminary hits are highly effective at restoring LFP recordings to a more wild-type state (Fig. 3b, Supplementary Table 3; progesterone, mifepristone, pargyline, promethazine, fluoxetine, and allopregnanolone). Pyrilamine may also show some efficacy.

### Validation of LFP analysis by deep behavioral phenotyping

**Fig. 4:**
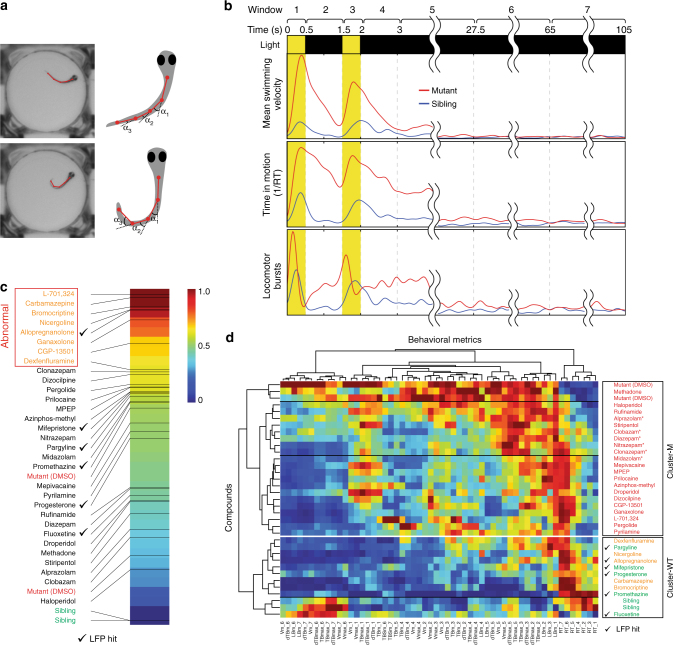
Deep behavioral phenotyping. **a** Automated image processing algorithms are used to locate the head and multiple points along the midline axis of the tail for each larva. Behavioral metrics are calculated based on these landmarks. **b** Seven temporal windows are defined relative to the onset of the seizure-inducing light stimulus: (1) first light pulse (0–0.5 s), (2) inter-pulse interval (0.5–1.5 s), (3) second light pulse (1.5–2 s), (4) early post-pulse 1 (2–3 s), (5) early post-pulse 2 (3–27.5 s), (6) early interictal (27.5–65 s), and (7) late interictal (65–105 s). Eight behavioral metrics are calculated using 40+ data points (*n* = 10+ larvae, each subjected to four independent light stimuli) per metric over all seven temporal intervals. Representative examples are shown for mean swimming velocity (**V**_m_), locomotor bursts (Burst_m_), and time spent in motion (RT) in untreated (1% DMSO) *scn1lab* mutants (red) and sibling controls (blue). **c** Behavioral fingerprints ranked based on Euclidean distance from sibling controls (green) during interictal periods (temporal windows 6 and 7). Compounds in the upper quartile (furthest from siblings) are presumed to have adverse side-effects on resting state behavior and are designated abnormal (orange text). Mutants are indicated in red text; hits based on LFP analysis are indicated by check marks. **d** A 56-component behavioral fingerprints are generated for each compound based on all eight behavioral metrics during all seven temporal windows. Each square represents the average value for that feature. Compounds and behavioral fingerprints are analyzed by hierarchical clustering to identify groups that produce similar behavioral outcomes. Cluster-M contains compounds with behavioral profiles similar to untreated mutants (red text) and Cluster-WT contains compounds with profiles similar to wild-type sibling controls (green text). Compounds in Cluster-WT that cause substantial alterations in resting state behavior (temporal windows 6 and 7) are indicated in orange text. Benzodiazepines are indicated with asterisk (*), hits based on LFP analysis are indicated by check marks

To confirm that brain activity pattern analysis identifies compounds with superior efficacy and fewer side effects, we carried out an in-depth behavioral assessment of all 31 preliminary hits using multiple independent metrics instead of a single-behavioral outcome (i.e., mean swimming velocity). In order to develop more sophisticated behavioral readouts, we used two complementary approaches. We first identified additional behavioral metrics that can be reliably detected and quantified from high-resolution video recordings of zebrafish larvae in multiwell plates (Fig. 4a). Metrics include mean swimming velocity (**V**_m_; pixels s^−1^), maximum swimming velocity (**V**_max_; pixels s^−1^), mean change in tail angle (dTB_m_; degrees s^−1^), maximum change in tail angle (dTB_max_; degrees s^−1^) mean tail bending angle (TB_m_; degrees), maximum tail bending angle (TB_max_; degrees), time spent at rest (RT; seconds), and number of locomotor bursts (LB_m_; defined as a transition from **V**=0 to **V** > 0). We then broke each behavioral metric down into temporal windows that were defined relative to the onset of the seizure-inducing light stimulus: (1) first light pulse (0–0.5 s), (2) inter-pulse interval (0.5–1.5 s), (3) second light pulse (1.5–2 s), (4) early post-pulse 1 (2–3 s), (5) early post-pulse 2 (3–27.5 s), (6) early interictal (27.5–65 s), and (7) late-interictal (65–105 s) (Fig. 4b).

To verify that in-depth behavioral metrics can reliably distinguish between mutants and siblings, we examined each metric using a large number (*n* = 40+) of DMSO-treated controls and our standard light stimulus parameters (Fig. 1a). Although individual behavioral metrics from single larvae show considerable variation—presumably due to the complex and stochastic nature of the neurological processes underlying photosensitivity and locomotor response—average metrics derived from multiple larvae exposed to multiple stimuli exhibit clear and robust patterns (Supplementary Fig. 10). As expected, most behavioral metrics in mutants undergo a dramatic change from baseline almost immediately after the onset of the light stimulus (Fig. 4b). Not surprisingly, wild-type siblings also initiate locomotor responses when subjected to light stimuli, although these differ substantially from mutants in both magnitude and overall temporal progression.

We rescreened all 31 preliminary hits at 4-h post exposure using deep behavioral phenotyping and the same assay parameters as the preliminary screen (Fig. 1a). An average activity profile was created for each compound by combining all recorded light stimulus events from all larvae (*n* = 40+) and normalizing all features. The result is a unique behavioral fingerprint made up of 56 values (eight behavioral metrics broken down into seven temporal windows) for each compound. Although behavioral fingerprints from untreated *scn1lab* mutants and sibling controls differ dramatically, the strongest differences are observed during the temporal windows encompassing the light stimulus itself and the subsequent seizure resolution period (from *t* = 0 s through *t* = 27.5 s). Behavioral metrics show fewer differences during the early and late interictal periods (*t* = 27.5–105 s), indicating that the *snc1lab* mutation does not substantially alter the mean behavioral profile in the absence of induced seizures. This becomes clear when all compounds are ranked based on Euclidean distance from siblings using only metrics from interictal temporal windows 6 and 7 (Fig. 4c). In this analysis, 19 out of 31 preliminary hits fall further from the wild-type end of the spectrum than untreated mutants, suggesting they alter relatively normal interictal behaviors and may have undesirable side effects that could give rise to false positives (e.g., sedation) or indicate other off-target concerns. We therefore flagged compounds ranked in the upper quartile of the interictal behavioral spectrum (i.e., those furthest from siblings) as abnormal.

**Fig. 5:**
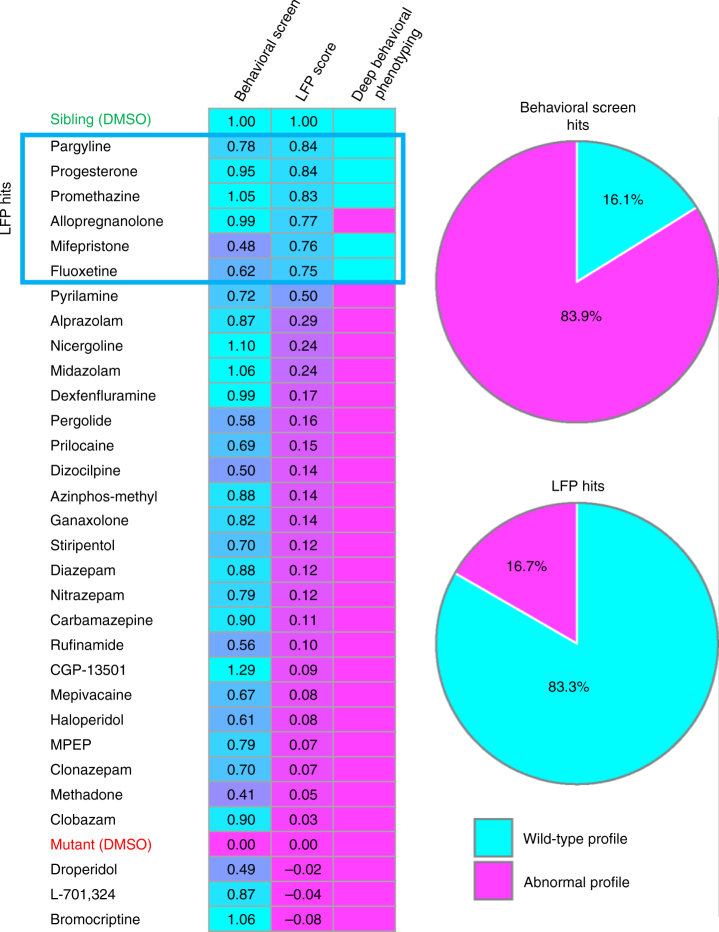
Brain activity pattern screening substantially reduces the false-positive rate. (Left) All compounds are ranked based on composite LFP scores. Compounds with a wild-type activity profile based on deep behavioral phenotyping (right column) are indicated in cyan; those with an abnormal profile are indicated in magenta. Top hits based on composite LFP scores are indicated in the blue box. (Right) Classification based on deep behavioral phenotyping (*n* = 10+ larvae, each subjected to four independent light stimuli) of all hits from the preliminary (single-metric) behavioral screen (*n* = 8+ larvae, each subjected to four independent light stimuli) and the LFP-complexity screen (*n* = 5–11 per compound). 26 out of 31 hits (83.9%) identified in the preliminary behavioral screen exhibit significant behavioral abnormalities when evaluated in detail. In contrast, only 1 out of 6 hits (16.7%) based on the composite LFP score has similar behavioral abnormalities

We then assessed behavioral fingerprints in detail by performing hierarchical clustering (MATLAB clustergram function, MathWorks, Natick, MA) based on Euclidean distance using Ward’s linkage algorithm^45^. On the resultant dendrogram, siblings and untreated mutants are located on highly divergent clusters (designated “Cluster-WT” and “Cluster-M”, respectively), indicating that the behavioral profiles of these two groups are strikingly different (Fig. 4d). The most effective compounds based on composite LFP scores all produce behavioral fingerprints that localize to Cluster-WT, confirming that brain activity pattern (BAP) analysis is a powerful tool for accurately assessing in vivo efficacy (Fig. 4d; LFP hits are indicated by a check mark). Several compounds with highly abnormal interictal behavioral profiles also localize to Cluster-WT, suggesting that behavioral side effects can indeed mimic AED activity and are likely responsible for many false positives in locomotor activity screens. Importantly, all of our top hits based on BAP analysis cluster with sibling controls in deep behavioral phenotyping and only one (allopregnanolone) exhibits abnormal interictal behavior, confirming that our approach, unlike screens based on simple locomotor metrics, reliably eliminates false positives while simultaneously avoiding false negatives (Fig. 5). Additionally, we observed that most of the structurally and mechanistically related benzodiazepines (5/6; 83%) co-localize to a single subcluster of the dendrogram, suggesting that behavioral fingerprints may prove useful for sorting neuroactive compounds into biologically meaningful groups in addition to assessing therapeutic endpoints (Fig. 4d). The benzodiazepine subcluster is located on Cluster-M along with untreated mutants, in agreement with our LPF data showing that benzodiazepines, at least at the concentrations used in our screen, are not highly effective at restoring brain activity to a more wild-type state.

## Discussion

Epilepsy impacts approximately 65 million people worldwide, with an annual incidence in the United States and in Europe of ~55 per 100,000. One in twenty-six people will develop epilepsy during their lifetime^46^. Although existing AEDs control seizures effectively in many patients, 30–40% remain refractory to treatment and develop chronic epilepsy. Even those who achieve adequate seizure control frequently experience undesirable cognitive and behavioral side effects^47^. For these reasons, the discovery of new therapeutics and alternative druggable targets remains a high priority. For over 60 years most in vivo AED screening has relied on behavioral readouts in rodent models. Unfortunately, rodents are poorly suited for large compound screens and behavioral readouts typically reduce complex neurological events to a few easily observed parameters. In this report, we show for the first time that direct analysis of brain activity patterns can be incorporated into a seizure model that is suitable for large-scale, high-throughput drug screening. Although we demonstrate our approach using a genetic model of epilepsy, the overall screening paradigm we propose is applicable to any disorder that alters normal brain activity patterns (BAPs).

Recently, multichannel platforms for simultaneous electrophysiological monitoring of multiple zebrafish larvae have been described^48^. However, unlike our high-throughput LFP platform, in which microelectrodes are inserted directly into the brain, these approaches detect activity from the exterior of the animals (as in EEG) resulting in smaller amplitude signals and an overall reduction in assay sensitivity. Their suitability for large-scale screening and novel drug discovery remains to be demonstrated. The superior sensitivity of our platform means that are able to decompose and analyze brain activity patterns during interictal periods in addition to detecting more obvious high-amplitude seizure-like events. This flexibility allows us to evaluate compounds based on two metrics: overall seizure frequency and interictal LFP signal complexity. Our results represent the first time that a high-throughput multichannel electrophysiology platform has been tested on more than a handful of compounds and cross-validated using in-depth behavioral assays.

Our approach to brain activity pattern analysis in the zebrafish *scn1lab* model is interestingly reminiscent of the recent work on adaptive deep brain stimulation. In Parkinson’s disease, exaggerated beta frequency oscillations and synchrony in the subthalamic nucleus correlate with reduced LFP complexity, which is a highly predictive metric of motor impairment in patients^49^. Application of deep brain stimulation during pathological beta oscillations reduces abnormal activity patterns and synchrony, and leads to clinical improvement^50^. Exactly why LFP complexity in Parkinson’s disease is linked to impairment remains to be determined, but signal complexity may be an indication of the information carrying or processing capability of the brain.

Based on both LFP pattern analysis and in-depth behavioral profiling, 6 of our 31 preliminary hits show clear efficacy in controlling seizure-like activity in *scn1lab* mutants, indicating a false-positive rate on the order of ~80% for simple behavioral screens. Previous behavior-based screens for AEDs have reported similar false-positive rates when preliminary hits are validated^14, 19^. This large number of false positives suggests that behavioral readouts based on single parameters (like mean swimming velocity) are highly susceptible to off-target effects that mimic desired therapeutic outcomes. Drugs with anesthetic, sedative, or related properties that suppress locomotor activity without correcting underlying pathologies are one likely source of false positives. Consistent with this, among our preliminary behavioral hits that failed in subsequent LFP screening were two local anesthetics (prilocaine and mepivacaine; both sodium channel blockers) and a number of compounds with well-documented sedative effects, including eight GABA_A_ receptor positive allosteric modulators (Supplementary Table 2). Although GABA_A_ receptor modulators, including benzodiazepines, are used to manage diverse types of epilepsies including DS, their therapeutic utility is often limited because of well-known side effects including sedation and cognitive impairment^51^. Much of the activity seen for benzodiazepines in our preliminary screen appears to be due to sedation rather than antiepileptic activity, since none were particularly effective in either LFP assays (Supplementary Table 3) or deep behavioral phenotyping (Fig. 4d).

Although both diazepam (a benzodiazepine) and stiripentol (a structurally novel modulator of the GABA_A_ receptor) failed to improve brain activity patterns in our hands, previous publications suggest that they are capable of reducing spontaneous electrographic seizures in *scn1lab* mutant zebrafish. However, both compounds have only been evaluated at extremely high concentrations (1 mM) in zebrafish^14^. In contrast, we screened them at substantially lower levels (10–20 μM) based on results from our preliminary toxicity assessment, which revealed a noticeable reduction in touch response at concentrations as low as 100 μM. This suggests that higher concentrations may produce significant side effects in addition to anti-seizure activity. To determine whether we could replicate published results using our platform and algorithms, we retested both diazepam and stiripentol at 100 and 200 μM. Under these conditions, we do indeed observe a significant reduction in the number of spontaneous seizure-like events in mutant larvae, however this is not accompanied by a corresponding improvement in the ICA complexity score (Supplementary Table 4, Supplementary Fig. 9). Additionally, when wild-type sibling controls are exposed to diazepam and stiripentol at these higher concentrations, we observe both a dramatic drop in their ICA complexity scores as well as significant behavioral side effects (Supplementary Table 4; Supplementary Fig. 11). Based on these data, we conclude that the antiepileptic activity of diazepam and stiripentol in *scn1lab* mutant larvae occurs only at concentrations that cause considerable off-target side effects. In the case of stiripentol, which has received orphan drug status for the treatment of DS, it should be noted that it is often combined with other AEDs clinically. At least some of its therapeutic activity is thought to arise from inhibition of their metabolism rather than from its own activity at the GABA_A_ receptor^52^. Such combinatorial activity is not studied in our screen, which only uses individual compounds.

The top hits from our LFP screen represent a remarkably diverse group of compounds. They include a first-generation antihistamine (promethazine), two pregnane steroids (progesterone and allopregnanolone), a selective serotonin reuptake inhibitor (fluoxetine), a selective monoamine oxidase-B inhibitor (pargyline), and a synthetic C19 norsteroid (mifepristone). A second antihistamine (pyrilamine, also known as mepyramine) shows marginal activity. Significantly, all five of the best compounds based on deep behavioral phenotyping are included among our top six LFP hits (Fig. 4d). This suggests a remarkably robust correlation between brain activity patterns and behavioral outcomes (Fig. 5).

Further validating our findings, one of our top hits (fluoxetine; a selective serotonin reuptake inhibitor), has recently been reported to cause a marked reduction in seizures in an adult woman with DS^53^. Additionally, recent studies using the zebrafish *scn1lab* model have shown that a number of other serotonergic modulators—including lorcaserin, trazodone, GR 46611, and TCB-2—are effective in suppressing seizures^54, 55^. Although these compounds were not included in our screen, their activity is consistent with what we observe for the serotonergic modulator fluoxetine. In particular, much like fluoxetine, trazodone’s antidepressant activity is thought to arise from inhibition of serotonin reuptake^56^. Somewhat surprisingly, we did not observe significant efficacy for either dexfenfluramine or clemizole (both putative serotonin modulators) in our LFP screen in spite of the fact that both have been identified in previous *scn1lab* screens^19, 57^ and fenfluramine is used clinically for treating DS^58–61^. As with diazepam and stiripentol, these discrepancies most likely stem from differences in dosing. We tested dexfenfluramine at 100 μM over the course of 4 h, whereas previous studies in zebrafish have used either higher concentrations (500 μM)^19^ or longer exposure times (24 h)^57, 62^. Similarly, we screened clemizole at a concentration of 10 μM based on our preliminary toxicity assessment, whereas previous studies have only reported anti-seizure activity at 300+ μM (for a 30-min exposure) and at 100 μM (for a 90-min exposure)^55^. As with diazepam and stiripentol, retesting clemizole at higher concentrations reduces the number of spontaneous seizures but fails to improve the ICA complexity score (Supplementary Table 4).

One potentially interesting class of compounds picked up by our screen is a structurally related group of C21 (pregnane) steroids. Our chemical library contained three members of this class (allopregnanolone, ganaxolone, and progesterone), all of which were among the top hits in our preliminary behavioral screen. Progesterone subsequently tested positive for anti-seizure activity based on both its composite LFP score and deep behavioral phenotyping, while allopregnanalone (3α-hydroxy-5α-pregnan-20-one) appeared to be highly effective based on its LFP score but showed some behavioral side effects. Among our top hits, allopregnanolone was the most effective at improving the seizure component of the LFP score (seizure score = 0.93) but the least effective at improving the ICA complexity component (ICA score = 0.69; Supplementary Table 3). Interestingly, although allopregnanolone’s effect on interical metrics (temporal windows 6 and 7; Fig. 4c) caused us to flag it as abnormal in deep behavioral phenotyping, it still clustered with sibling controls (Fig. 4d) due to its efficacy at reducing abnormal light-triggered ictal behaviors (temporal windows 1–5). This outcome—strong anti-seizure activity in combination with abnormal locomotor activity and a relatively poor ICA score—is reminiscent of what we observed with high concentrations of diazepam and stiripentol (Supplementary Table 4). Taken together, these data suggest allopregnanolone many possess both anti-seizure activity and off-target side effects.

Allopregnanolone is an endogenous pregnane neurosteroid that is synthesized in vivo by 5α-reduction of progesterone^63, 64^. It has been shown to exhibit potent anticonvulsant effects in diverse animal models^63^ and clinically in patients with super-refractory status epilepticus^65^ and appears to be less subject to anticonvulsant tolerance than benzodiazepines^66, 67^. Unlike progesterone, allopregnanolone is not believed to have activity at nuclear steroid hormone receptors^64, 68^. Conversely, progesterone itself is not a GABA_A_ receptor modulator, but it can be metabolized to allopregnanolone and other related compounds. The anti-seizure effects of progesterone may therefore occur through its conversion to allopregnanolone, as has been shown in other animal models^69^. However, the time course of this effect is remarkably rapid based on our data, with light-induced seizure-like locomotor activity dropping to 31% of baseline within 45 min of application (Supplementary Data 1) and spontaneous LFP-detected seizure frequency decreasing to 27% of baseline in a comparable timeframe (Supplementary Table 3, seizure frequency, 45 min column). Based on our results, allopregnanolone and compounds with related pregnane skeletons warrant further investigation as therapeutics for DS.

## Methods

### Fish maintenance

All procedures on live animals were approved by the Massachusetts Institute of Technology Committee on Animal Care. The scn1lab^s552^ line (also known as *double indemnity* or *didy*) has been described previously^14^. The scn1lab^sa16474^ line was obtained from the Zebrafish International Resource Center (Eugene, OR; ZIRC catalog # ZL9291.15). Both lines were outcrossed to the wild type TAB-14 line (ZIRC catalog #ZL1438) prior to analysis. Adult zebrafish were maintained under standard laboratory conditions and larvae were staged as described^70^. Fertilized eggs were generated by crossing heterozygous adults and raised on a 12 h light/12 h dark cycle at 28 °C in E3 medium (5 mM NaCl, 0.17 mM KCl, 0.33 mM CaCl_2_, 0.33 mM MgSO_4_, pH 7.2).

### Compound screening

At 7 dpf, homozygous mutant larvae were identified based on the presence of dispersed melanosomes, which give rise to a darkly pigmented phenotype^37^, and single larvae were distributed into individual wells of flat-bottomed 96-well microplates (MultiScreen 96-well Transport Receiver Plate, Millipore, Billerica, MA) in a volume of 100 μL of E3 medium per well. Microplates with larvae were placed into the enclosed, light-controlled imaging chamber of our automated video tracking system (described in detail below) and allowed to adapt to the dark environment for 20 min. Following adaptation, but prior to the application of test compounds, locomotor activity was assessed in response to light stimuli to determine the baseline activity level. Each light stimulus was delivered by a computer controlled LED and consisted of two 500 ms light pulses separated by 1 s of darkness. Locomotor activity was calculated based on response to four light stimuli, each separated by 2 min of darkness (Fig. 1a).

Stocks of all test compounds were prepared at 10 mM in 100% dimethyl sulfoxide (DMSO), aliquoted, and stored at −20 °C (see Supplementary Data 1 for CAS number, supplier, and catalog number). On the day of the experiment, 2× working stocks of each compound were prepared in E3 medium and the DMSO concentration was adjusted to 2%. A volume of 100 μL of the 2× working stock was added to each well immediately after acquisition of the baseline locomotor recording (*n* = 8–10 larvae per compound), resulting in the indicated screening concentrations (Supplementary Data 1) and a final DMSO concentration of 1%. Following compound addition, additional locomotor activity recordings were acquired at beginning at 45- min, 2 h, and 4 h post exposure using the same light stimuli parameters that were used for the baseline recordings (Fig. 1a).

For preliminary compound screening, the mean swimming velocity of each larva at each assay time point (baseline, 45 min, 2 h, and 4 h post exposure) was calculated based on total locomotor activity recorded during the 5 s intervals following each of the four light stimuli. All post-exposure velocities were normalized to baseline activity level for each treatment group and a correction factor was applied to compensate for a slight upward drift in locomotor activity that was consistently observed in DMSO-only controls at all post-exposure time points. For any given post-exposure recording session, this correction factor was equal to the mean velocity of the untreated (i.e., DMSO-only) control group during the baseline recording session divided by the mean velocity of the same untreated control group during the post-exposure recording session.

### Automated video tracking system

Locomotor activity was recorded using a custom-built video tracking system comprising a monochrome CCD camera (Manta G-033; Allied, Exton, PA) fitted with a motorized close-focusing macro video lens (Zoom 7000 lens system, Navitar, Rochester, NY), a near-IR longpass filter (LP800-52, MidOpt, Palatine, IL), a motorized stage (H101A; Prior, Rockland, MA) and a IR, whitelight LED illuminator (BX 06 06 WHI/IR, Advance illumination, Rochester, VT). The system was surrounded by a custom-made light-tight optical table enclosure (1 m × 1 m × 0.4 m) and mounted on an optical breadboard base (Newport Corporation, Irvine, CA). The IR/LED illumination was controlled by an Arduino Mega 2560 microcontroller board (digital output range from 0.0 to 5.0 V; Adafruit Industries, New York, NY).

### High-throughput LFP recording platform

The recording chamber of the high-throughput LFP platform is made of two 1 × 2 × 8 cm aluminum bars placed in parallel using two 4 cm screws, directly jointing two aluminum bars. Glass capillaries (1.1 mm inner diameter; 1.5 mm outer diameter; BF150-110, Sutter Instrument Company, Novato, CA) serve to hold agar-embedded zebrafish larvae. These are arranged in a parallel configuration and inserted through equally spaced mounting holes drilled along one side of the recording chamber. In order to seal the recording chamber, each mounting hole is equipped with a water tight 1.2 mm gasket that fits tightly around each capillary. On the opposite side of the recording chamber, glass recording electrodes are inserted through a second series of mounting holes precisely co-centered with those holding the glass capillaries. Recording electrodes are made by pulling a 1 mm outer diameter capillary (BF100-78-10, Sutter Instrument Company), which is filled with 1 M chloride solution. The electrodes are held using a 1 mm water tight gasket to fully waterproof the whole chamber. A Ag/Cl wire (64–1320, Harvard Apparatus, Holliston, MA) is then placed inside each electrode, each wire is connected to a 16-channel preamplifier (RHD2216, Intan Technologies, Los Angeles, CA), and the preamplifier is connected to a low-power digital acquisition chip (RHD2000, Intan Technologies; Fig. 2c). The signal from acquisition board is recorded using Intan MATLAB GUI software (MATLAB 13, Mathworks, Natick, MA). The data are then denoized and analyzed to obtain independent components using custom MATLAB code as described below.

### Dual-layer agarose embedding

In order to immobilize and precisely position zebrafish within the glass capillaries for extended LFP recording sessions, we devised a process through which larvae can be embedded in a cylinder of 1.3% ultra-low gelling temperature agarose (which solidifies at 25 °C; A2576, Sigma) surrounded by shell of 2% low gelling temperature agarose (which solidifies at 55 °C; A0701, Sigma; Fig. 2a, b). Unlike most immobilization protocols used for LFP recording in zebrafish larvae, ours does not require any paralytic agent. The ultra-low gelling temperature core allows larvae to be safely added to the agarose while in a liquid state without being exposed to excessive temperatures. The more rigid low gelling temperature agar shell strengthens and supports the inner core, allowing it to be inserted into the glass capillary and ensuring that the larvae are fully immobilized. Embedding is accomplished by first transferring larvae into a solution of liquid 1.3% ultra-low gelling temperature agarose, which is then poured into a 20 mL syringe. The 20 mL syringe is then inserted into a 60 mL syringe filled with 2% low gelling temperature agarose. The 20 mL syringe is capped with an 18-gauge blunt tip dispensing needle and the 60 mL syringe is capped with a 16-gauge needle. We then simultaneously extrude both agarose solutions into a room temperature bath containing E3 medium (Fig. 2a). Following embedding, larvae are transferred to the glass capillaries of the LFP recording platform and recording electrodes are advanced into the forebrains using miniaturized screws. The electrical resistance and the average voltage on each electrode is monitored as it penetrates the forebrain. Advancement is halted when resistance decreases to 3 MΩ and the average noise is <0.2 mV RMS. If resistance deviates from the initial value by more than 50% over the course of the recording, the sample is excluded from further analysis since it indicates the electrode may have shifted position or become damaged. Unless otherwise noted, all electrophysiological screening was done using 5–11 larvae per compound/condition (with an average of 8.3 per condition).

### Automated seizure detection

Automated seizure detection is accomplished using XGBoost, an open-source machine learning system for gradient tree boosting^71^, to classify electrophysiological signals into seizure and non-seizure classes using higher-order statistical moments as features. Similar approaches using higher order statistics have been used successfully to classify EEG signals^40^. Training and test data sets for building and evaluating our gradient boosting algorithm are generated using 10 min LFP recordings from 20 *scn1lab* mutants, which are subjected to our standard light-stimulus protocol in order to trigger seizures on demand at known time points (Fig. 1a). The amplified signal is filtered using a first-order 3 kHz lowpass anti-aliasing filter and sampled at a rate of 3k samples per second. The time series signal is then filtered using a second-order Butterworth bandpass 0.5 Hz to 1 kHz filter and divided into 30 s windows with 20 s of overlap between each consecutive window. Windows corresponding to light stimuli are coded as “seizure” intervals; all others are coded as “non-seizure” intervals. For each 30 s interval, we extract five statistical metrics from the time-varying amplitude of the signal: mean, variance, skewness, kurtosis, and fourth power mean. The feature vector for each 30 s window consists of 15 total features: 5 features from the window itself and well as 5 from each of the two preceding partially overlapping windows. We use the features from 80% of the windows as a training data set for our gradient boosting algorithm. We use 20 decision trees each with a maximum depth of 5 to accomplish this. The remaining 20% of the windows are used as a validation data set to evaluate the performance of the trained algorithm. Similar to the previous work using higher-order statistics to detect seizures^40^, our algorithm gives very high seizure detection accuracy (~95%).

### Independent component analysis

We use an automated single-channel ICA algorithm previously described^42^ to find independent components corresponding to separate sources before and after seizure onset. We start with LFP recordings performed under our standard light stimulus parameters (Fig. 1a). The amplified signal is filtered as described above and divided into 30 s vectors with 80% overlap between each consecutive vector. The vectors are arranged into a data matrix *X*, which is a superposition of independent vectors. Assuming $W = E\left[ {XX^t} \right]$ is the autocorrelation matrix, the correlation between samples is removed to generate a white spectrum random process as follows:

$${\mathrm{\Omega }} = W^{ - 1/2}{X}W^{ - 1/2}$$

Now consider ${\mathrm{\Omega }} = \mathop {\sum }\nolimits \alpha _i\beta _i$, where $\beta _i$ is the independent vector and $\alpha _i$ is the scaling factor. The first independent vector is obtained by optimizing the fourth moment of time series^72^:

$${\mathrm{min}}_{\beta _1:||\beta _1|| = 1}{\sum} {\left[ {\beta _1^T\left. {\left( {{\mathrm{\Omega }} - {\bar{\mathrm \Omega }}} \right)} \right]} \right.^4}$$

The residual is then calculated by subtracting the first component signal using ${\mathrm{\Omega }}_{i}{\mathrm{ = \Omega }}_{{i} - 1} - \left[ {\beta _{i - 1}{\mathrm{\backslash \Omega }}_{i - 1}} \right]\beta _{i - 1}$, where ${\mathrm{\Omega }}_1{\mathrm{ = \Omega }}$.The residual is used to calculate the next independent vector as:

$${\mathrm{min}}_{\beta _i:||\beta _i|| = 1}{\sum} {\left[ {\beta _i^T\left( {{\mathrm{\Omega }}_i - {\bar{\mathrm \Omega }}} \right)} \right]^4}$$

The *i*th independent component (IC_*i*_) of the signal over time is calculated by ${\mathrm{IC}}_i(t) = \beta _i\backslash {\mathrm{\Omega }}({t})$, where $\beta _i$ is the solution of the optimization and where ${\mathrm{\Omega }}({i})$ is the *i*th 30 s vector and ${\bar{\mathrm \Omega }}$ is the average of ${\mathrm{\Omega }}({t})$.

### Automated deep behavioral phenotyping

Segmentation of the microplate wells is done with a snake algorithm^73^. The known rough position of the microwell plate is used as the initial guess for the snake. Larvae are tracked using a background subtraction approach to extract moving features. An empty background image is first created by taking the maximum of all frames in the video stream. This background image is then subtracted from each frame in the recording session. Larvae tails are enhanced with a Gabor filter^74, 75^ and a threshold is used to generate a binary image of all larvae. The position of the head is found by detecting the local maxima of the low-pass filtered foreground image^76^. To speed up the algorithm, we perform a correlation between the detected object in each well with the object in the previous frame. If the correlation is above a predetermined threshold the larva is considered to be stationary and the result from previous frame is used. A skeletonization is done on the binary object followed by a distance transform from the head constrained by the binary skeleton. The point furthest away from the head is considered to be the tip of the tail. We next find the centerline of the larval body and tail. The shortest path is calculated from the head to the tip of the tail with the Gabor filtered enhanced image as weights using the MATLAB Accurate Fast Marching plugin^77, 78^. Five equally spaced vertex points are positioned along the detected centerline (shortest path) from the head to the tail tip.

We use the five centerline points to calculate several features. All features are broken down into temporal windows as described in the text and both mean and maximum feature values are calculated for every window. The mean swimming velocity (**V**_m_; pixels s^−1^) and maximum swimming velocity (**V**_max_; pixels s^−1^) are calculated based on the distance traveled by the head of the larva. The tail angle is calculated for each vertex point and the absolute values of all points are summed together. These values are used to calculate the mean tail bending angle (TB_m_; degrees) and the maximum tail bending angle (TB_max_; degrees). Mean change in tail angle (dTB_m_; degrees s^−1^) and maximum change in tail angle (dTB_max_; degrees s^−1^) are based on the sum of change in tail angle for each vertex point. When there is no change in tail angle, a fish is considered to be at rest and these frames are used to calculate time spent at rest (RT; seconds). The last feature we calculate is the number of locomotor bursts (LB_m_;). This metric is based on the number of transitions from rest to active tail movement that occur within a given temporal window. All feature metrics are combined to create a behavioral fingerprint for each compound. Fingerprints are automatically assessed using MATLAB’s clustergram function (MathWorks, Natick, MA) to perform hierarchical clustering based on Euclidean distance using Ward’s linkage algorithm^45^ and to generate heat maps.

### Code availability

Core source code for LFP analysis, seizure detection, and basic video tracking of larvae is available online from GitHub (https://github.com/rezaie99/NC-17-6337).

### Data availability.

Any data sets generated and analyzed during the current study that are not contained within the manuscript are available from the corresponding author on reasonable request.

## Electronic supplementary material

Supplementary Information

Description of Additional Supplementary Files

Supplementary Data 1

Supplementary Movie 1
