# Supplementary material for: Brain activity patterns in high-throughput electrophysiology screen predict both drug efficacies and side effects
Source: Nat Commun. 2018 Jan 15;9:219. doi: 10.1038/s41467-017-02404-4 (PMC5768723; doi:10.1038/s41467-017-02404-4)
Supplement: Supplementary file 1 — Supplementary Information [file 41467_2017_2404_MOESM1_ESM.pdf]

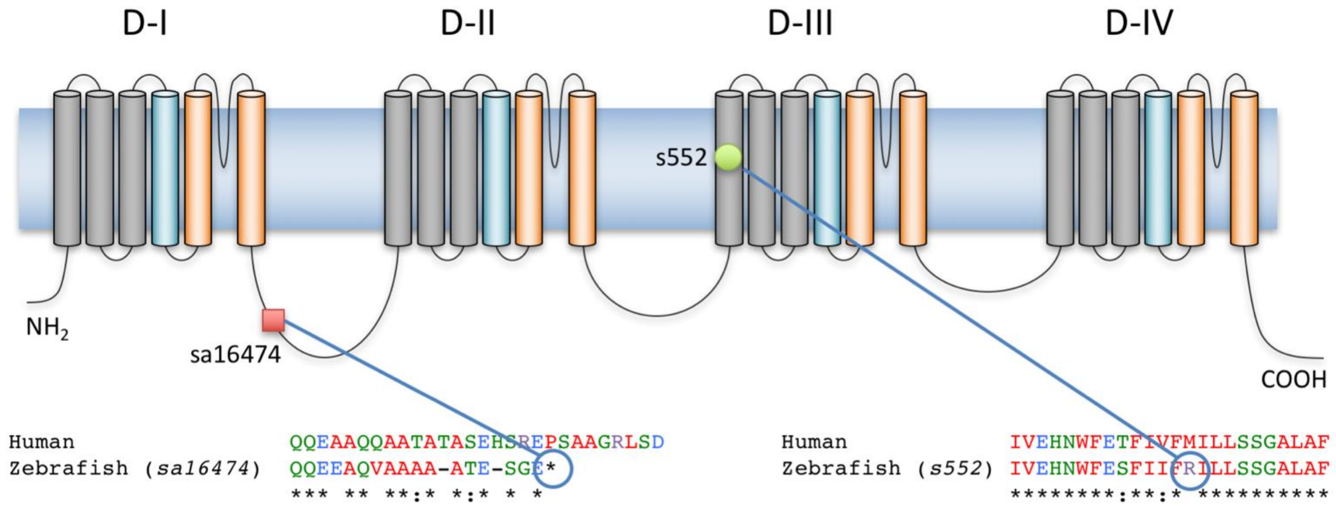

Supplementary Figure 1: Location of the s552 and sa16474 point mutations in the Na_V sodium channel alpha subunit. Cylinders represent individual transmembrane segments making up the four ion channel domains (D-1 through D-IV; gray: S1–S3 segments; blue: S4 voltage-sensing segment; orange: S5–S6, ion pore region). Green circle, missense mutation (p.Met1208Arg); red square, truncation (p.Tyr462*). Alignments of the human (SCN1A) and zebrafish (Scn1lab) protein sequences are shown flanking the site of both mutations.

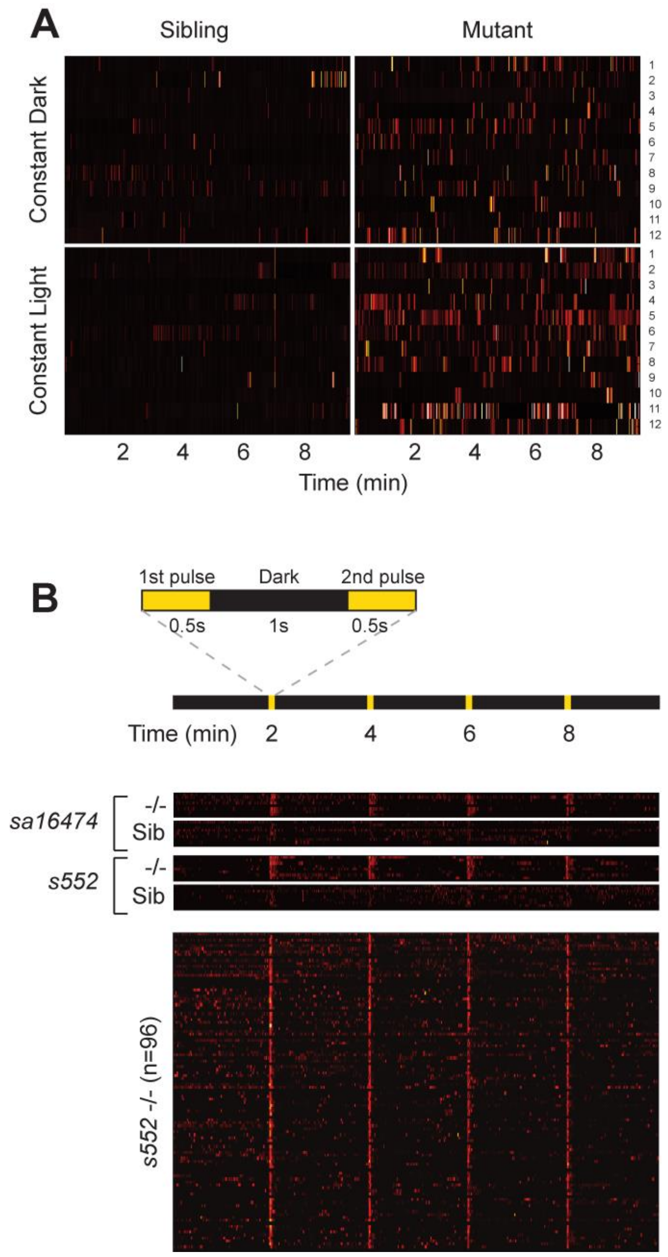

Supplementary Figure 2: Zebrafish larvae with mutations in *scn1lab* exhibit light-induced seizures. (a,b) Locomotor activity plots of individual larvae recorded for 10 minutes in a 96-well plate at 7 dpf using an automated tracking platform. Periods of movement are indicated in color; periods of rest are indicated in black. **(a)** Locomotor activity of *scn1lab*^{s552} homozygous mutant larvae and age-matched sibling controls under constant dark (top panels) and constant light (bottom panels). **(b)** Light stimuli trigger bursts of seizure-like activity in mutants but not sibling controls. (top) Light stimuli are applied every two minutes in an otherwise dark environment. Each stimulus consists of two consecutive 500 millisecond light pulses separated by 1 second of dark. (center) Light-triggered locomotor activity in homozygous mutants (-/-) and sibling controls (Sib) from *scn1lab*^{s552} and *scn1lab*^{sa16474} lines. (bottom) Light-triggered locomotor activity is a highly robust phenotype and is observed in nearly all in mutant larvae in all wells of a 96-well plate.

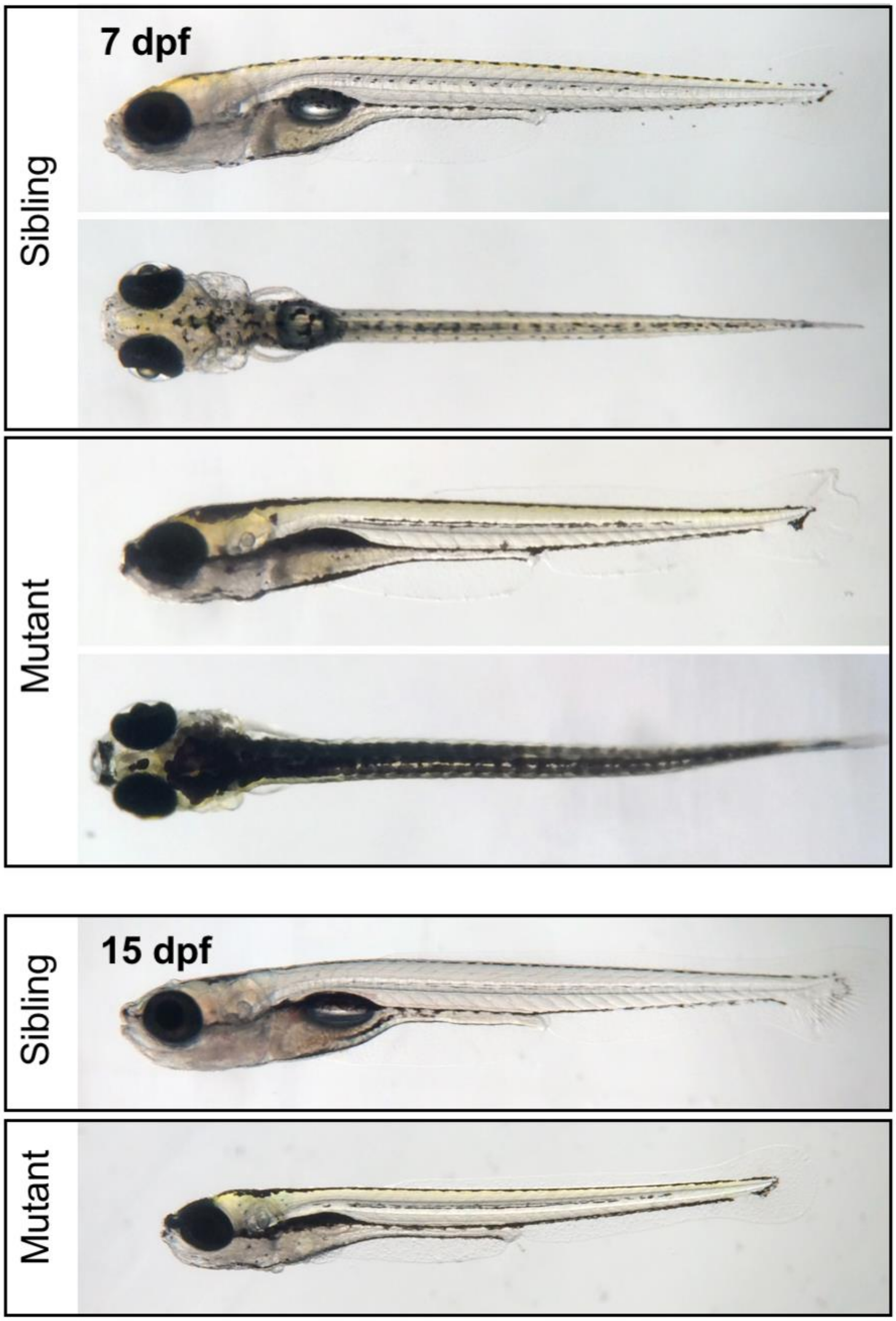

Supplementary Figure 3: Visible phenotypes in the *scn1lab*^{sa16474} line. (Top) Lateral and dorsal views of an sa16474 homozygous mutant and an age-matched sibling control larva at 7 dpf. Mutant larvae exhibit dark coloration due to dispersed melanosomes and fail to inflate their swim bladders. (Bottom) Lateral view of an sa16474 homozygous mutant and an age-matched sibling control larva shown at the same magnification at 15 dpf. Mutants fail to thrive and show elevated levels of mortality beginning in the second week of development (survival of mutants at 14 dpf=32%, n=28; survival of siblings at 14 dpf=95%, n=39).

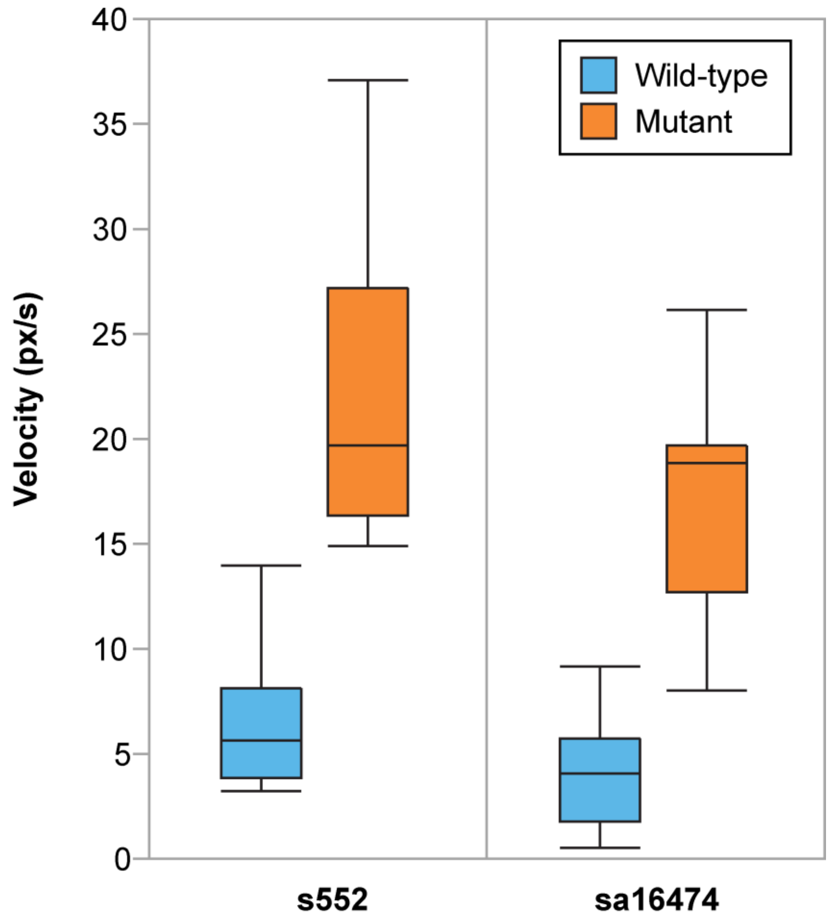

Supplementary Figure 4: Light stimuli trigger seizure-like activity in *scn1lab* mutant larvae from two independent lines. Box-and-whisker plots show mean swimming velocity (pixels per second) for homozygous mutants (orange) and age-matched sibling controls (blue) obtained from the same clutch. Larvae were recorded in a 96-well plate using an automated tracking platform (s552: n=8 per genotype; sa16474: n=15 per genotype; each larva was subjected to 4 independent light stimuli). Each light stimulus consists of two consecutive 500 millisecond light pulses separated by 1 second of darkness. Mean swimming velocities are calculated during the 5 second periods immediately following each light pulse. Tops and bottoms of each box represent the 1st and 3rd quartiles. Whiskers are drawn from the ends of the interquartile ranges (IQR) to the outermost data point that falls within ±1.5 times the IQR. The line in the middle of each box is the sample median.

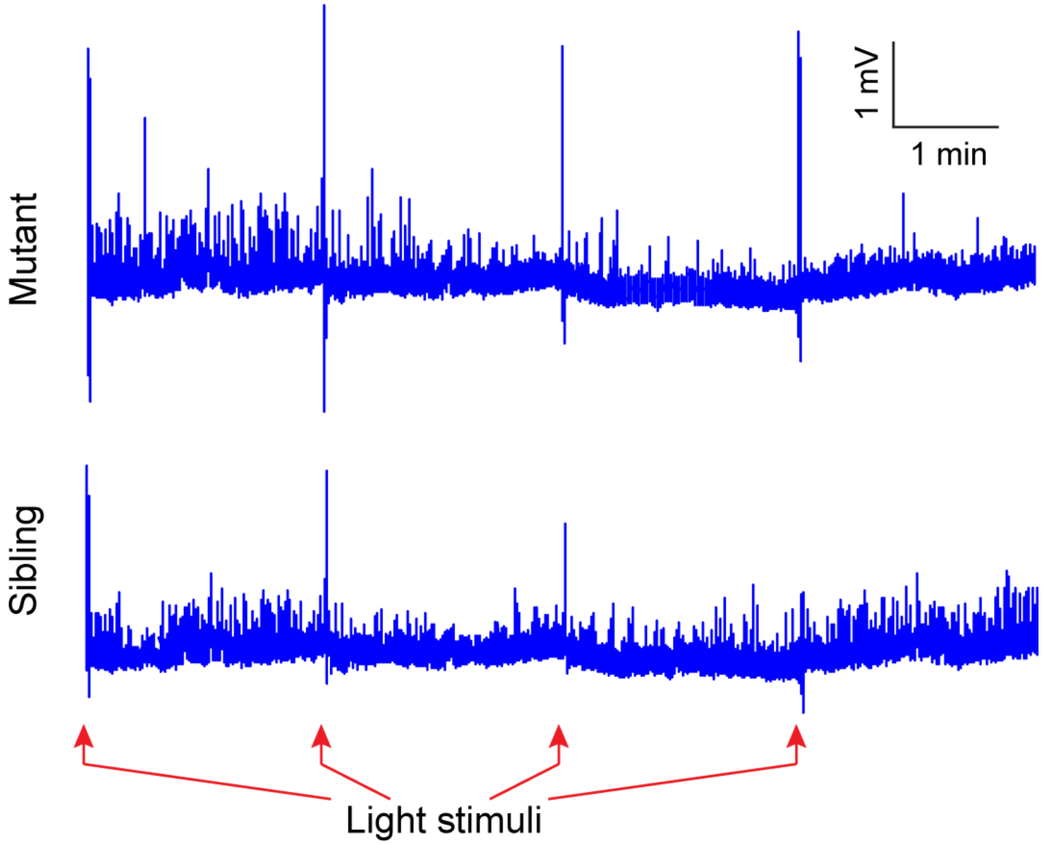

Supplementary Figure 5: **Response to light stimuli in mutants and sibling controls.** Representative 10 minute LFP recordings from an *scn1lab* mutant and an age-matched sibling control showing response to multiple light stimuli (see **Fig. 1a** for stimulus parameters).

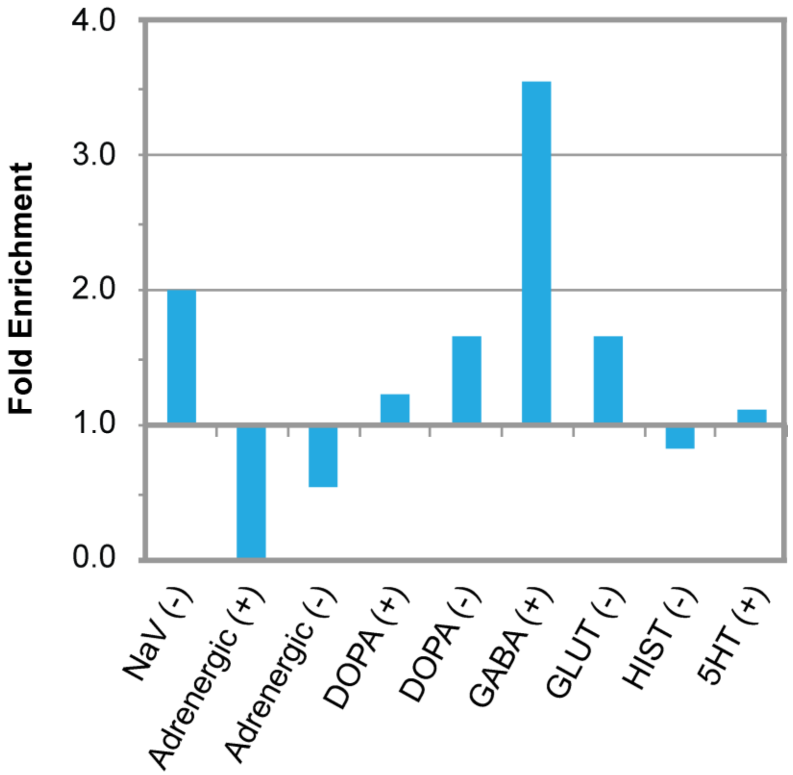

Supplementary Figure 6: Results of preliminary screen based on locomotor activity. For each class of drugs, the graph indicates fold enrichment over what would be expected by chance alone among the preliminary hits.

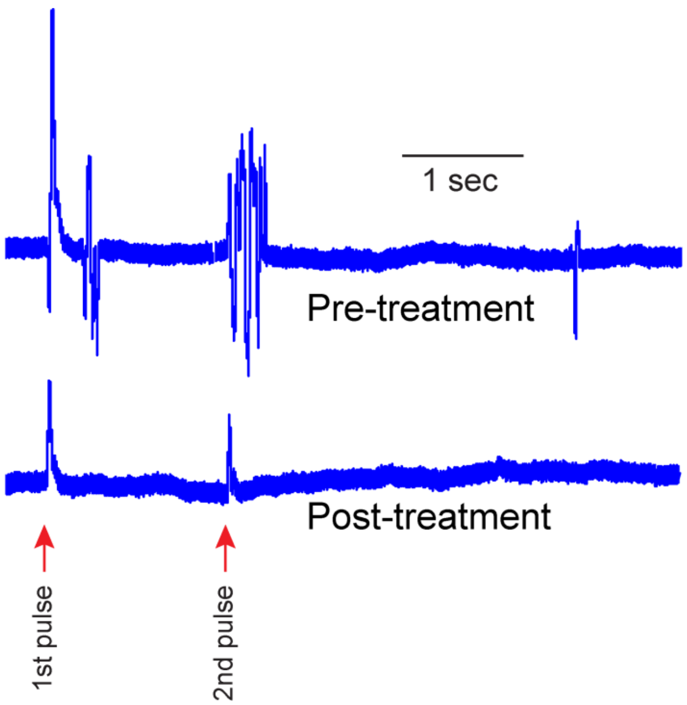

Supplementary Figure 7: Effective compounds reduce light-triggered seizures in addition to spontaneous seizures. Representative LFP recordings from an *scn1lab*^{s552} mutant larva during light stimulation (see Fig. 1a for stimulus parameters). The untreated recording was made immediately prior to compound application and the treated recording was made 2 hours after addition of progesterone.

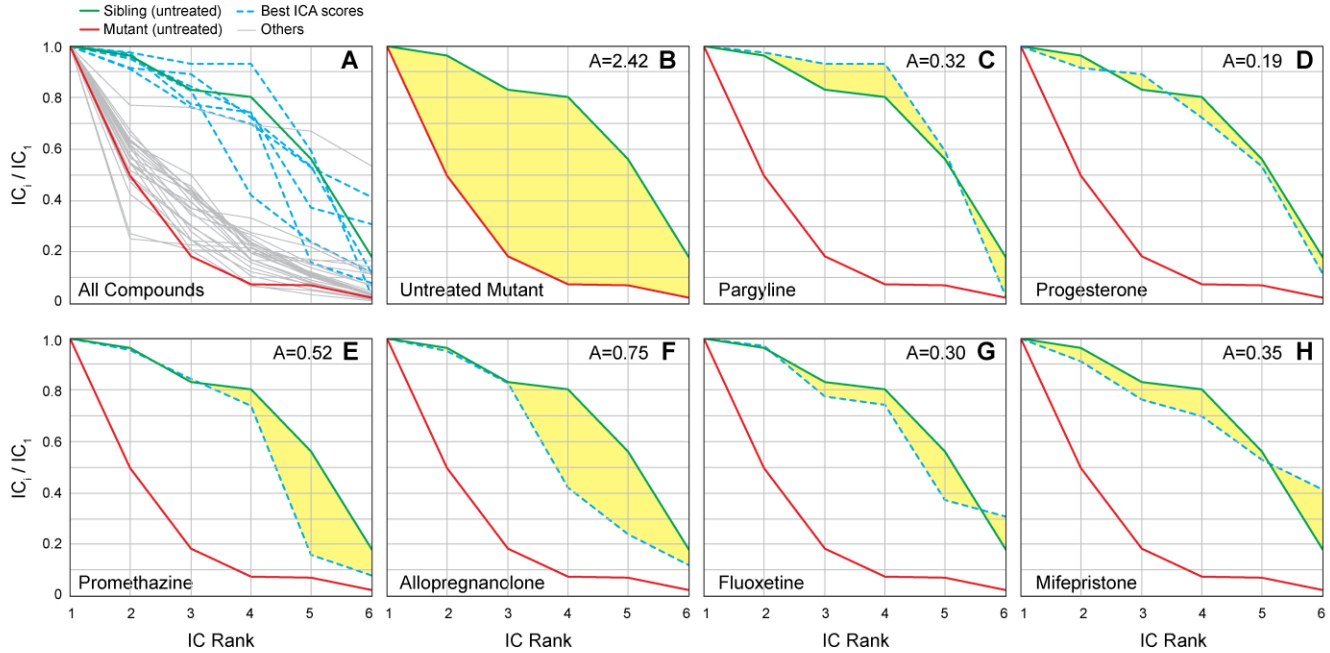

Supplementary Figure 8: Independent component analysis shows reduced LFP complexity in *scn1lab* mutants. Plots show the relative contribution of the top 6 independent components (ICs) in wild-type sibling controls (1% DMSO, green line), untreated mutants (1% DMSO, red line), and compound-treated larvae beginning at ~240 minutes post-exposure. All ICs are normalized to the top ranked IC (IC_i/IC_1). (**a**) IC profiles from all 31 preliminary hits. Top hits based on composite LFP scores are indicated with dashed blue lines, all others are in gray. Complex LFPs from siblings and from mutants treated with effective compounds show reduced attenuation in the lower-ranked ICs. (**b-h**) Divergence from the normal (i.e. sibling control) IC profile can be quantified by calculating the total area (A; arbitrary units) separating the sibling IC profile from the experimental IC profile of interest, as indicated by yellow shading. (**b**) The IC profile of untreated *scn1lab* mutants diverges substantially from that of sibling controls. (**c-h**) Effective compounds shift the IC profile in mutant larvae toward sibling controls.

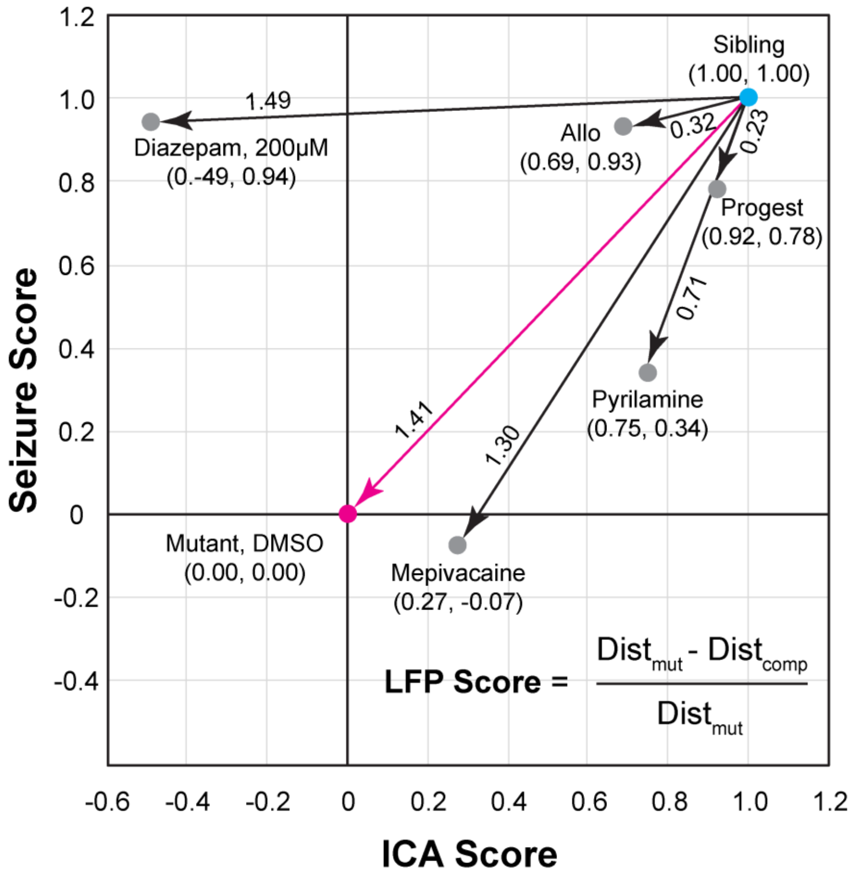

Supplementary Figure 9: Composite LFP scores. Seizure scores and ICA scores are combined by using each variable to specify a point on a scatterplot. For each compound, the composite LFP score is then calculated as follows: $(Dist_{mut} - Dist_{comp})/Dist_{mut}$, where $Dist_{mut}$ is the Euclidean distance between sibling controls and untreated *scn1lab* mutants and $Dist_{comp}$ is the distance between sibling controls and mutants exposed to the compound of interest.

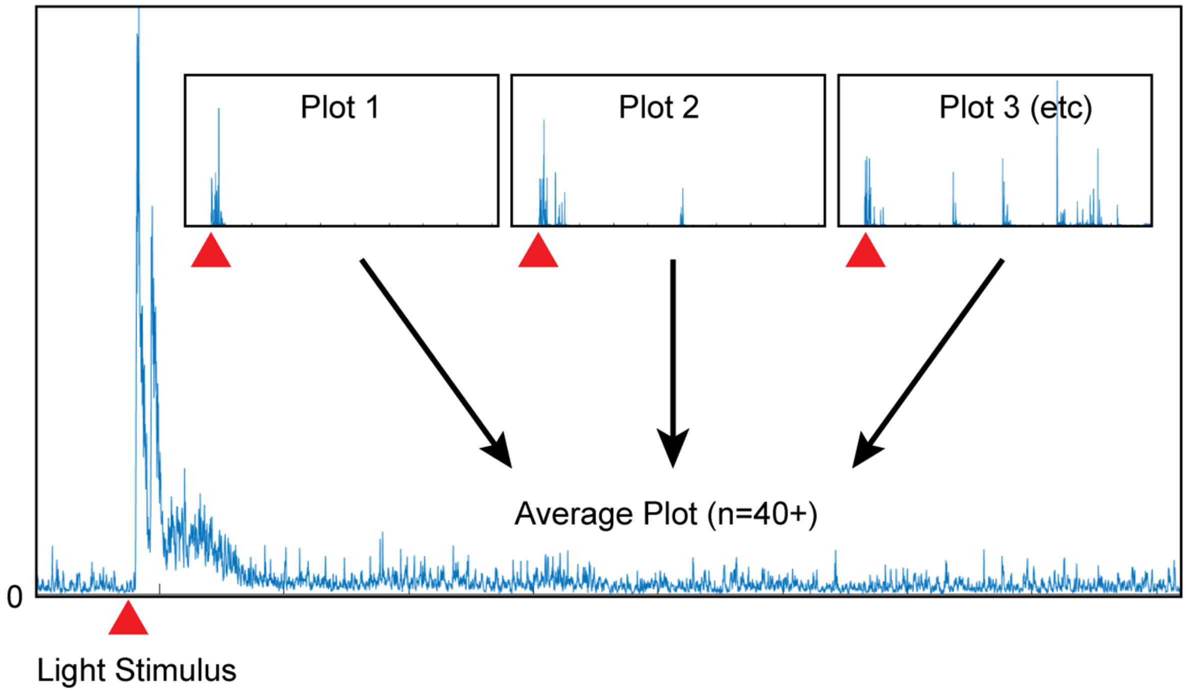

Supplementary Figure 10: **Deep behavioral profiling.** The effect of each compound is evaluated at 4 hours post-exposure on 10 or more larvae in multiwell plates by applying 4 seizure-inducing light stimuli, resulting in 40+ data points per compound (see Fig. 1a for stimulus parameters). For each compound, an average activity plot is then created for each metric by combining all 40+ data points. Red arrows indicate the onset of the light stimulus.

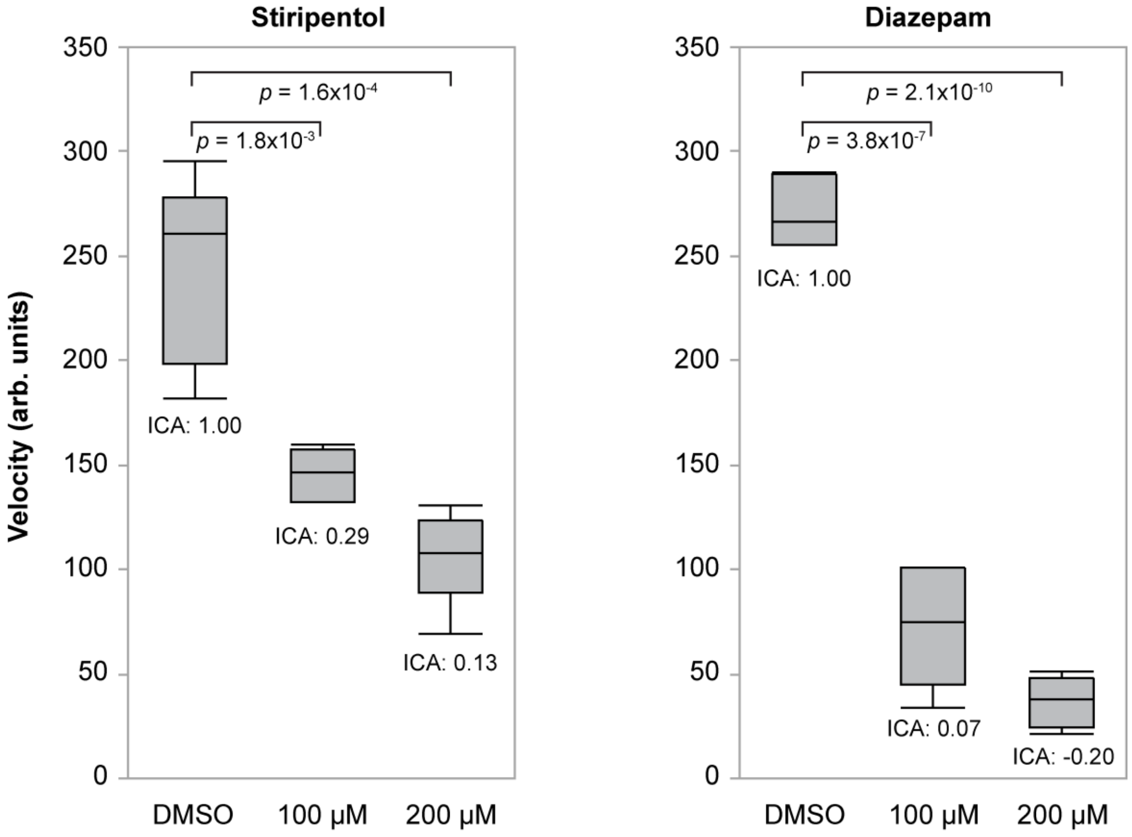

Supplementary Figure 11: Effect of stiripentol and diazepam at high concentrations on wild-type locomotor activity and ICA score. Box-and-whisker plots showing mean swimming velocity (arbitrary units) for wild-type sibling controls 4-hours post-exposure to 1% DMSO alone or stiripentol or diazepam at the indicated concentrations (in 1% DMSO). Larvae were recorded in a 96-well plate using an automated tracking platform (n=6 larvae were used per condition; each larva was exposed to 4 independent light stimuli consisting of two consecutive 500 millisecond light pulses separated by 1 second of darkness). Mean swimming velocities were calculated over the full 10-minute recording session. Tops and bottoms of each box represent the 1st and 3rd quartiles. Whiskers are drawn from the ends of the interquartile ranges (IQR) to the outermost data point that falls within ±1.5 times the IQR. The line in the middle of each box is the sample median. Statistical significance was determined by Welch's *t*-test. Corresponding ICA scores from LFP analysis are shown under each box-and-whisker plot.

Supplementary Table 1: Drugs with established clinical activity in DS patients.

Compound	45 min	2 h	4 h	Dravet	Primary Pathway(s)	References
Midazolam	29	23	24	Effective	GABA (+)	Ceulemans (2011)
(+)-Fenfluramine	27	29	33	Effective	Serotonin (+)	Ceulemans et al. (2012)
Carbamazepine	45	37	26	Contra	Na+ channels (-)	Chiron (2011), Miller et al. (2014)
Clobazam	34	34	40	Effective	GABA (+)	Shi et al. (2016)
Diazepam	34	40	39	Effective	GABA (+)	Miller et al. (2014)
Clonazepam	52	48	52	Effective	GABA (+)	Miller et al. (2014), Shi et al. (2016)
Stiripentol	47	50	55	Effective	GABA (+)	Chiron (2011), Miller et al. (2014)
Fluoxetine	87	57	26	Effective	Serotonin (+)	Meador (2014)
Rufinamide	74	48	60	Effective	Na+ channels (-)?	Kim et al. (2013)
Phenytoin	89	87	76	Contra	Na+ channels (-)	Miller et al. (2014),
Vigabatrin	86	89	83	Contra	GABA (+)	Miller et al. (2014)
Valproic acid	86	82	95	Effective	GABA (+)	Miller et al. (2014)
Oxcarbazepine	101	79	85	Contra	Na+ channels (-)	Gelisse et al. (2004), Xu et al. (2014)
Zonisamide	104	81	88	Effective	Na+/Ca2+ channels (-)?	Shi et al. (2016)
Phenobarbital	101	103	85	Contra	GABA (+)	Chiron (2011), Xu et al. (2014)
Lamotrigine	107	106	108	Contra	Na+ channels (-)?	Chiron (2011), Miller et al. (2014)
Topiramate	125	112	160	Effective	GABA (+), Glutamate (-)	Chiron (2011), Miller et al. (2014)

Controls comprise eleven drugs that are either commonly used to treat DS or have shown efficacy in human studies (effective; green) and six drugs that have been reported to worsen seizures in patients with DS (contraindicated; red).

Supplementary Table 2. Preliminary hits based mean swim velocity in two *scn1lab* mutant lines

Line	s552			sa16474					
Compound	45min	2h	4h	45min	2h	4h	Reported anticonv.	Primary Pathway(s)	Mechanism Details
Siblings	32	28	28	29	52	36	NA	NA	Age-matched sibling controls
(+)-Fenfluramine	27	29	33	49	37	36	Yes	Serotonin (+)	Serotonin releasing agent; may also release norepinephrine and dopamine
Allopregnanolone	26	28	36	34	35	45	Yes	GABA (+)	Positive allosteric modulator of the GABAA receptor; endogenous pregnane neurosteroid
Alprazolam	43	39	33	27	20	22		GABA (+)	Positive allosteric modulator of the GABAA receptor; benzodiazepine
Azinphos-methyl	41	39	34	38	43	28		Acetylcholine (+)	Acetylcholinesterase inhibitor; organophosphate insecticide
Bromocriptine	34	24	17	26	15	8		Dopamine (+)	Preferential agonist of the dopamine D2 receptor; additional agonistic activity on 5-HT and α-adrenergic receptors
Carbamazepine	45	37	26	32	9	5	Yes	Na+ channels (-)	Inhibits Na+ channels
CGP-13501	7	2	17	5	0	0		GABA (+)	Positive allosteric modulator of the GABAB receptor
Clobazam	34	34	40	48	28	48	Yes	GABA (+)	Positive allosteric modulator of the GABAA receptor; benzodiazepine
Clonazepam	52	48	52	30	18	21	Yes	GABA (+)	Positive allosteric modulator of the GABAA receptor; benzodiazepine
Diazepam	34	40	39	38	34	43	Yes	GABA (+)	Positive allosteric modulator of the GABAA receptor; benzodiazepine
Dizocilpine	93	60	40	113	83	76	Yes	Glutamate (-)	Non-competitive antagonist of the NMDA receptor
Droperidol	89	66	41					Dopamine (-)	Antagonist of the dopamine D2 receptor; additional antagonistic activity on α1-adrenergic receptor
Fluoxetine	87	57	26	74	37	26	Yes	Serotonin (+)	Selective serotonin reuptake inhibitor
Ganaxolone	39	47	41	64	71	88	Yes	GABA (+)	Positive allosteric modulator of the GABAA receptor; synthetic analog of allopregnanolone
Haloperidol	78	51	41	102	105	115		Dopamine (-)	Antagonist of the dopamine D2 receptor; additional targets may include 5-HT2, NMDA, and α-adrenergic receptors
L-701,324	44	35	36	39	4	13	Yes	Glutamate (-)	Antagonist of the NMDA glutamate receptor
Mepivacaine	66	49	42	120	80	84		Na+ channels (-)	Inhibits Na+ channels; local anesthetic
Methadone	94	75	45	68	64	66		Opiates (+)	Agonist of the μ-opioid receptor; may block the NMDA glutamate receptor; synthetic opioid
Midazolam	29	23	24	19	21	16	Yes	GABA (+)	Positive allosteric modulator of the GABAA receptor; benzodiazepine
Mifepristone	89	69	41	90	46	18		Progesterone (-), Glucocorticoids (-)	Antagonist of the progesterone and glucocorticoid receptors
MPEP	48	42	43	34	28	36	Yes	Glutamate (-)	Antagonist of the metabotropic glutamate receptor 5
Nicergoline	33	17	16	68	50	69		NE/EPI (-)	Antagonist of the α1-adrenergic receptor
Nitrazepam	52	44	36	35	20	19	Yes	GABA (+)	Positive allosteric modulator of the GABAA receptor; benzodiazepine
Pargyline	56	43	36	35	43	18		Monoamines (+)	Inhibitor of monoamine oxidase B; may also inhibit monoamine oxidase A
Pergolide	40	58	79	44	44	61		Dopamine (+)	Agonist of the dopamine D2 receptor; additional agonistic activity at dopamine D3, α-adrenergic, and 5-HT receptors
Prilocaine	58	53	42	101	68	72		Na+ channels (-)	Inhibits Na+ channels; local anesthetic
Progesterone	31	28	38	23	25	29	Yes	Progestogen (+)	Agonist of the nuclear progesterone receptor; antagonist of the mineralocorticoid receptor; precursor of neurosteroids such as allopregnanolone
Promethazine	36	27	14	60	38	18		Histamine (-)	Antagonist of the histamine H1 receptor; additional antagonistic activity at the muscarinic acetylcholine receptor
Pyrilamine	79	43	24	105	67	56		Histamine (-)	Antagonist of the histamine H1 receptor
Rufinamide	74	48	60	77	50	63	Yes	Unclear [Na+ channels (-)?]	Prolongs inactive state of Na+ channels; exact therapeutic mechanism unclear
Stiripentol	47	50	55	47	44	49	Yes	GABA (+)	Agonist of the GABAA receptor

Supplementary Table 3. Brain activity pattern analysis

Compound	IC1-2	IC2-3	IC3-4	IC4-5	IC5-6	Total Area	ICA Score	45 min	120 min	240 min	Seizure Score
Sibiling (DMSO)	0.00	0.00	0.00	0.00	0.00	0.00	**1.00**	0.00	0.00	0.00	**1.00**
Pargyline	0.01	0.05	0.11	0.08	0.07	0.32	**0.87**	0.30	0.14	0.16	**0.82**
Progesterone	0.02	0.03	0.04	0.06	0.05	0.19	**0.92**	0.29	0.25	0.19	**0.78**
Promethazine	0.00	0.00	0.03	0.23	0.25	0.52	**0.79**	0.81	0.29	0.09	**0.90**
Allopregnanolone	0.01	0.01	0.19	0.35	0.19	0.75	**0.69**	0.38	0.20	0.06	**0.93**
Mifepristone	0.03	0.06	0.09	0.07	0.11	0.35	**0.86**	0.69	0.57	0.27	**0.69**
Fluoxetine	0.00	0.03	0.06	0.12	0.08	0.30	**0.88**	0.50	0.31	0.29	**0.67**
Pyrilamine	0.10	0.13	0.09	0.06	0.23	0.60	**0.75**	0.42	0.38	0.58	**0.34**
Alprazolam	0.18	0.38	0.49	0.51	0.29	1.85	**0.23**	0.90	0.71	0.57	**0.35**
Nicergoline	0.21	0.41	0.50	0.52	0.29	1.92	**0.20**	0.80	0.66	0.63	**0.28**
Midazolam	0.16	0.36	0.48	0.51	0.29	1.80	**0.25**	0.85	0.66	0.68	**0.22**
Dexfenfluramine	0.21	0.44	0.47	0.40	0.19	1.71	**0.29**	1.10	1.00	0.82	**0.06**
Pergolide	0.17	0.34	0.47	0.50	0.21	1.69	**0.30**	0.90	0.82	0.84	**0.04**
Prilocaine	0.23	0.50	0.60	0.58	0.32	2.23	**0.08**	1.04	0.78	0.68	**0.22**
Dizocilpine	0.16	0.37	0.49	0.50	0.27	1.78	**0.26**	0.92	0.69	0.84	**0.03**
Azinphos-methyl	0.20	0.39	0.49	0.53	0.30	1.91	**0.21**	1.01	0.78	0.81	**0.08**
Ganaxolone	0.15	0.35	0.48	0.50	0.29	1.77	**0.27**	0.97	0.82	0.85	**0.02**
Stiripentol	0.22	0.44	0.51	0.52	0.30	1.99	**0.18**	1.03	0.75	0.81	**0.07**
Diazepam	0.21	0.45	0.56	0.55	0.31	2.08	**0.14**	0.91	0.85	0.79	**0.10**
Nitrazepam	0.17	0.38	0.48	0.50	0.29	1.82	**0.25**	0.93	0.73	0.87	**0.01**
Carbamazepine	0.19	0.48	0.66	0.63	0.35	2.31	**0.04**	0.79	0.83	0.71	**0.18**
Rufinamide	0.20	0.43	0.49	0.43	0.21	1.76	**0.27**	0.92	0.97	0.91	**-0.04**
CGP-13501	0.27	0.56	0.63	0.58	0.32	2.37	**0.02**	0.98	0.83	0.73	**0.17**
Mepivacaine	0.14	0.36	0.54	0.51	0.20	1.76	**0.27**	1.05	0.90	0.94	**-0.07**
Haloperidol	0.17	0.37	0.52	0.55	0.31	1.92	**0.21**	1.04	0.77	0.90	**-0.03**
MPEP	0.18	0.42	0.51	0.47	0.26	1.83	**0.24**	0.97	0.93	0.94	**-0.08**
Clonazepam	0.36	0.66	0.60	0.52	0.30	2.43	**-0.01**	0.90	0.73	0.75	**0.15**
Methadone	0.35	0.66	0.61	0.53	0.30	2.45	**-0.01**	0.87	0.86	0.76	**0.13**
Clobazam	0.18	0.46	0.62	0.60	0.34	2.20	**0.09**	0.98	1.04	0.89	**-0.02**
Mutant (DMSO)	0.23	0.56	0.69	0.61	0.32	2.42	**0.00**	1.05	0.88	0.87	**0.00**
Droperidol	0.25	0.54	0.58	0.49	0.24	2.09	**0.13**	0.95	1.07	1.01	**-0.15**
L-701,324	0.24	0.51	0.59	0.56	0.32	2.21	**0.08**	1.01	0.83	1.01	**-0.15**
Bromocriptine	0.21	0.52	0.68	0.62	0.34	2.37	**0.02**	1.12	0.90	1.01	**-0.16**

The IC areas represent the area (arbitrary units) separating the IC profiles of wild-type sibling controls and compound-treated *scn1lab* mutants. ICA scores are calculated as described in the manuscript from 45 minute LFP recording sessions beginning ~240 minutes post-exposure. Seizure frequencies are determined using an automated seizure detection algorithm and are broken down into three intervals: 0-45 minutes post-exposure (45 min), 55-120 minutes post-exposure (120 min), and 130-240 minutes post-exposure (240 min). Between each interval, larvae are subjected to our standard light-stimulus protocol. Seizure scores are calculated as described in the manuscript and are based on seizure frequency during the final interval (130-240 minutes post-exposure). Composite LFP scores combine the ICA score and the seizure score (n=5-11 per compound), as described in the manuscript and illustrated in **Supplementary Fig. 9**. All compounds are ranked based on their composite LFP score. Top hits based on composite LFP scores are indicated in the blue box.

Supplementary Table 4. Analysis of diazepam, stiripentol, and clemizole at high concentrations

		IC Areas							Seizure frequency				
	Compound	IC1-2	IC2-3	IC3-4	IC4-5	IC5-6	Total Area	ICA Score	45 min	120 min	240 min	Seizure Score	Composite LFP Score
	Mutant (DMSO)	0.16	0.46	0.60	0.49	0.26	1.96	0.00	0.99	0.94	0.91	0.00	0.00
	Sibling (DMSO)	0.00	0.00	0.00	0.00	0.00	0.00	1.00	0.00	0.00	0.00	1.00	1.00
Mutants	Mutant-Diazepam (10µM)	0.18	0.40	0.52	0.52	0.30	1.91	0.03	0.92	0.90	0.82	0.10	0.06
Mutants	Mutant-Diazepam (100µM)	0.30	0.61	0.65	0.58	0.33	2.47	-0.26	0.72	0.21	0.09	0.90	0.11
Mutants	Mutant-Diazepam (200µM)	0.40	0.77	0.75	0.65	0.36	2.93	-0.49	0.48	0.13	0.05	0.94	-0.06
Mutants	Mutant-Stiripentol (20µM)	0.13	0.27	0.37	0.38	0.19	1.34	0.32	1.04	0.79	0.89	0.03	0.16
Mutants	Mutant-Stiripentol (100µM)	0.28	0.53	0.53	0.48	0.25	2.07	-0.05	0.70	0.36	0.17	0.81	0.24
Mutants	Mutant-Stiripentol (200µM)	0.32	0.59	0.58	0.50	0.26	2.25	-0.15	0.55	0.14	0.04	0.96	0.19
Mutants	Mutant-Clemizole (10µM)	0.11	0.29	0.41	0.43	0.26	1.49	0.24	0.90	0.62	0.44	0.52	0.36
Mutants	Mutant-Clemizole (100µM)	0.23	0.47	0.55	0.52	0.30	2.07	-0.05	0.86	0.40	0.26	0.71	0.23
Mutants	Mutant-Clemizole (200µM)	0.28	0.57	0.61	0.52	0.29	2.26	-0.15	0.76	0.31	0.11	0.88	0.18
Siblings	Sibling-Diazepam (10µM)	0.07	0.19	0.32	0.35	0.22	1.15	0.42					
Siblings	Sibling-Diazepam (100µM)	0.20	0.40	0.48	0.48	0.26	1.82	0.07					
Siblings	Sibling-Diazepam (200µM)	0.34	0.62	0.58	0.51	0.31	2.36	-0.20					
Siblings	Sibling-Stiripentol (20µM)	0.01	0.02	0.14	0.19	0.05	0.42	0.79					
Siblings	Sibling-Stiripentol (100µM)	0.07	0.21	0.42	0.46	0.24	1.40	0.29					
Siblings	Sibling-Stiripentol (200µM)	0.21	0.39	0.43	0.42	0.26	1.71	0.13					
Siblings	Sibling-Clemizole (10µM)	0.01	0.04	0.17	0.21	0.07	0.50	0.74					
Siblings	Sibling-Clemizole (100µM)	0.05	0.20	0.42	0.45	0.24	1.37	0.30					
Siblings	Sibling-Clemizole (200µM)	0.19	0.38	0.45	0.43	0.26	1.71	0.13					

Low — High

Diazepam, stiripentol, and clemizole were evaluated at increasing concentrations (gray triangles) in wild-type sibling controls and *scn1lab* mutants at 5 dpf (n=6+ larvae per condition). The IC areas represent the area (arbitrary units) separating the IC profiles of sibling controls and compound-treated *scn1lab* mutants. ICA scores are calculated as described in the manuscript from 45 minute LFP recording sessions beginning ~240 minutes post-exposure. Seizure frequencies are determined using an automated seizure detection algorithm and are broken down into three intervals: 0-45-minutes post-exposure (45 min), 55-120-minutes post-exposure (120 min), and 130-240-minutes post-exposure (240 min). Between each interval, larvae are subjected to our standard light-stimulus protocol (each larva is subjected to 4 independent light stimuli). Seizure scores are calculated as described in the manuscript and are based on seizure frequency during the final interval (130-240 minutes post-exposure). Composite LFP scores combine the ICA score and the seizure score as described in the manuscript and illustrated in **Supplementary Fig. 9**.

Supplementary References

1 Ceulemans, B. *et al.* Successful use of fenfluramine as an add-on treatment for Dravet syndrome. *Epilepsia* **53**, 1131-1139, doi:10.1111/j.1528-1167.2012.03495.x (2012).

2 Meador, K. J. Seizure Reduction with Fluoxetine in Dravet Syndrome. *Epilepsy Behav Case Rep* **2**, 54-56, doi:10.1016/j.ebcr.2014.03.001 (2014).

3 Kim, S. H. *et al.* Short-term efficacy and tolerability of rufinamide adjunctive therapy in children with refractory generalised epilepsy. *Epileptic Disord* **15**, 49-54, doi:10.1684/epd.2013.0557 (2013).

4 Xu, X. *et al.* Early clinical features and diagnosis of Dravet syndrome in 138 Chinese patients with SCN1A mutations. *Brain Dev* **36**, 676-681, doi:10.1016/j.braindev.2013.10.004 (2014).

5 Miller, I. O. & Sotero de Menezes, M. A. in *GeneReviews(R)* (eds R. A. Pagon *et al.*) (2014).

6 Chiron, C. Current therapeutic procedures in Dravet syndrome. *Dev Med Child Neurol* **53 Suppl 2**, 16-18, doi:10.1111/j.1469-8749.2011.03967.x (2011).

7 Shi, X. Y. *et al.* Efficacy of antiepileptic drugs for the treatment of Dravet syndrome with different genotypes. *Brain Dev* **38**, 40-46, doi:10.1016/j.braindev.2015.06.008 (2016).

8 Gelisse, P. *et al.* Worsening of seizures by oxcarbazepine in juvenile idiopathic generalized epilepsies. *Epilepsia* **45**, 1282-1286, doi:10.1111/j.0013-9580.2004.19704.x (2004).

9 Ceulemans, B. Overall management of patients with Dravet syndrome. *Dev Med Child Neurol* **53 Suppl 2**, 19-23, doi:10.1111/j.1469-8749.2011.03968.x (2011).
