# Supplementary material for: Brain activity patterns in high-throughput electrophysiology screen predict both drug efficacies and side effects
Source: Nat Commun. 2018 Jan 15;9:219. doi: 10.1038/s41467-017-02404-4 (PMC5768723; doi:10.1038/s41467-017-02404-4)
Supplement: Supplementary file 2 — Description of Additional Supplementary Files [file 41467_2017_2404_MOESM2_ESM.pdf]

File Name: **Supplementary Data 1**
Description: **Compound Library**

File Name: **Supplementary Movie 1**
Description: **Light stimuli trigger seizure-like activity in *scn1lab* mutant larvae**. Video showing light-triggered locomotor activity in *scn1lab*^{s552} homozygous mutants (left half of the plate) and age-matched sibling controls (right half of the plate). Recordings were performed in darkness using IR-illumination. The two dark wells in the bottom row contain a fluorescent dye that is sensitive to illumination in the visible spectrum and indicate the timing of the light stimuli.
